# A Computer-Assisted 3D Model for Analyzing the Aggregation of Tumorigenic Cells Reveals Specialized Behaviors and Unique Cell Types that Facilitate Aggregate Coalescence

**DOI:** 10.1371/journal.pone.0118628

**Published:** 2015-03-19

**Authors:** Amanda Scherer, Spencer Kuhl, Deborah Wessels, Daniel F. Lusche, Brett Hanson, Joseph Ambrose, Edward Voss, Emily Fletcher, Charles Goldman, David R. Soll

**Affiliations:** 1 Monoclonal Antibody Research Institute, Developmental Studies Hybridoma Bank, Department of Biology, University of Iowa, Iowa City, Iowa, 52242, United States of America; 2 Mercy Hospital System of Des Moines, Des Moines, Iowa, United States of America; University of Missouri-Columbia, UNITED STATES

## Abstract

We have developed a 4D computer-assisted reconstruction and motion analysis system, J3D-DIAS 4.1, and applied it to the reconstruction and motion analysis of tumorigenic cells in a 3D matrix. The system is unique in that it is fast, high-resolution, acquires optical sections using DIC microscopy (hence there is no associated photoxicity), and is capable of long-term 4D reconstruction. Specifically, a z-series at 5 μm increments can be acquired in less than a minute on tissue samples embedded in a 1.5 mm thick 3D Matrigel matrix. Reconstruction can be repeated at intervals as short as every minute and continued for 30 days or longer. Images are converted to mathematical representations from which quantitative parameters can be derived. Application of this system to cancer cells from established lines and fresh tumor tissue has revealed unique behaviors and cell types not present in non-tumorigenic lines. We report here that cells from tumorigenic lines and tumors undergo rapid coalescence in 3D, mediated by specific cell types that we have named “facilitators” and “probes.” A third cell type, the “dervish”, is capable of rapid movement through the gel and does not adhere to it. These cell types have never before been described. Our data suggest that tumorigenesis *in vitro* is a developmental process involving coalescence facilitated by specialized cells that culminates in large hollow spheres with complex architecture. The unique effects of select monoclonal antibodies on these processes demonstrate the usefulness of the model for analyzing the mechanisms of anti-cancer drugs.

## Introduction

Tumors develop in three dimensions in tissues. Therefore, *in vitro* models that allow tumor cells to form aggregates in three dimensions rather than in two dimensions on a flat substrate should, a priori, more accurately depict the *in vivo* process. Moreover, such models should provide a more accurate venue for analyzing interactions with, and the degradation of, the extracellular matrix [1–3], for investigating the involvement of normal cell types such as immune cells [4,5], fibroblasts [6] and endothelial cells [7] in tumorigenesis, for analyzing the dynamics of tumor cells during multiplication and aggregate coalescence [8,9], and for testing the effects of potential anti-tumorigenic agents on the aforementioned processes [10].

In the early 1990’s, tumor cells began to be cultured by embedding them in 3D transparent gels [11,12]. It soon became apparent that the propagation of tumor cells and the development of tumor cell aggregates were not only different from that of normal cells, but were also different from the behavior of tumor cells on two dimensional substrates [13,14]. Subsequently, a number of studies demonstrated the value of 3D models, especially for analyzing how tumor cell aggregates degrade the supporting 3D matrix [15–18].

In past studies using 3D models, tumor cell multiplication and the development of tumor cell aggregates were imaged with compound microscopy or confocal microscopy of living or fixed fluorescent preparations. Using the latter method, optical sections [19] were obtained and 3D reconstructions of fluorescing cells forming aggregates were generated [17,20,21]. However, this method was limited for analyses over time (i.e., 4D studies) due to photoxicity of both the fluorophore and the excitation light [22–25], as well as the limited speeds of synchronized optical sectioning. Pampaloni et al. [26] recently described a light sheet fluorescence microscope (LSFM) that allowed repeated optical sectioning of cells in an agarose gel that is purported to result in no phototoxicity based on measurements of cell cycle length [26]. However, there have been no dedicated computer-assisted systems described that pose no toxicity problems over a 30 day period, automatically optically section the same developing aggregates of cancer cells in 3D at short time intervals over extended time periods, reconstruct them at time intervals and motion analyze them. Given, as we shall demonstrate, that the formation of aggregates by tumor cells and subsequent aggregate coalescence occurs in 3D over a time frame of weeks, and involves specialized cell interactions that facilitate coalescence in a time frame of hours, a system was required that could obtain a set of optical sections in the Z-axis in a time interval of one minute. Furthermore, the process should be repeatable at time intervals as short as every 5 minutes, and continue for several weeks. Such a system would require methods to reconstruct not only the developing aggregates, but also single cells. If that system automatically converted the reconstructed aggregates and cells into 3D mathematical models, then it could automatically quantify a variety of motility, contour and coalescence parameters in 3D over time. And finally, such a system would have to accomplish these tasks employing optics that did not cause phototoxicity.

Here, we describe a system that fulfills these requirements. Moreover, the first application of this system has revealed that 3D preparations of tumorigenic cell lines and fresh tumor biopsies, but not cells from non-tumorigenic lines or normal fresh tissue, undergo aggressive coalescence, facilitated by the development and behavior of specialized cell types.

Computer-assisted technologies for obtaining optical sections of unstained cell preparations within short time intervals over a long time period during cell translocation and chemotaxis, and for quantitatively analyzing cell contour and behavior with time, have been available for almost two decades [27–34]. They were never, to our knowledge, applied by cancer researchers, except for studies of the behavior of individual cancer cells translocating on a 2D substrate [35]. One of the most advanced of these programs, the 3D Dynamic Image Analysis System (3D-DIAS) [31,33,36,37], was modified in 2002 to reconstruct and motion-analyze every cell division, including every nuclear division, during development of the transparent *Caenorhabditis elegans* embryo, through the 28-cell stage [37]. The system involved automatic collection over a 2.5 second period of a Z-series of 75 optical sections through a preparation using differential interference contrast (DIC) microscopy rather than fluorescent microscopy. This procedure was repeated at 5 second intervals over a 2 hour period. Because the embryonic cells of *C*. *elegans* are transparent, the optical sections obtained by DIC microscopy contained the perimeters of all cells, which were then autotraced using the software 3D-DIASemb [37]. DIC imaging had no toxicity and provided the speed necessary for optically sectioning a rapidly locomoting cell or developing embryo. Optical sections could be collected at video speeds of one per thirtieth of a second. The in-focus images in each optical section in the Z-axis could then be automatically or manually traced, and reconstructed as 3D images. A time sequence of whole cell images, aggregates, nuclei, filopodia, pseudopodia, and other subcellular components distinguishable by DIC microscopy, could be reconstructed in 3D at short time intervals over an extended time period with the only limitation being culture conditions. A sequence of such 3D reconstructions provided a dynamic movie that allowed visualization of the dynamics of filopodia, nuclei, cells and cell aggregates in 3D from any angle, and the planes and relative timing of each cell division. Because the perimeters of each subcellular component, cell or aggregate, were converted to mathematical models, over 20 3D parameters of motility and contour change could be computed over time and over 30 2D parameters could be computed through any plane (optical section) of the 3D image [27,28,33,34,37–39].

By modifying the previous 3D-Dynamic Image Analysis System (3D-DIAS) [29,36,37], we developed a specialized JAVA-based version, J3D-DIAS 4.1, dedicated to reconstructing and motion analyzing cancer cells, the aggregates they form, and the unique characteristic of coalescence between aggregates, in a clear gel, in this case Matrigel. The system, as applied here, provides reconstructions every 30 minutes of aggregates and selected cells in a 3D region of the gel (between 10 and 25 objects in a 446 by 335/μm field) over periods of 17 days or more. Application of this system has revealed a variety of unique behaviors in the process of cancer cell aggregation and has allowed us to identify for the first time two specialized cell types, the “facilitator” and “probe”. Both emerge from aggregates after 100 hours of incubation and facilitate coalescence. Control preparations of cells from a non-tumorigenic cell line or from fresh tissue underwent multiplication in Matrigel, but the subsequent cell aggregates neither coalesced nor formed the specialized cell types observed in a variety of tumorigenic cell lines or fresh tumor cell preparations. This 4D analysis has also revealed a third cell type, the “dervish”, which emerges from cancer cell preparations and translocates through aggregates and inter-aggregate spaces at extraordinarily high velocities in a swirling pattern, apparently playing no role in aggregate coalescence and exhibiting no cohesivity to aggregates. The challenge now will be to investigate whether the unique behaviors of tumorigenic aggregates multiplying in Matrigel; i.e., aggregate coalescence, the formation and emergence of specialized cell types and the developmental program that results in highly organized aggregate spheres with cell-free interiors, play roles in tumorigenesis *in vivo*. In addition, we have tested whether the model can be useful in assessing the role of anti-tumorigenic drugs. To this end, we have analyzed the aggregation process of cells of a tumorigenic cell line in the presence of two monoclonal antibodies (mAb), one against ß-1-integrin, which was previously demonstrated to normalize breast cancer cell aggregate formation in a 3D model [13,40,41] and one against α-3 integrin [42]. We show that the anti-ß-1-integrin mAb did not inhibit cancer cell multiplication, but did completely block aggregate coalescence and the formation of both facilitator and probe cells, and that the anti-α-3 integrin mAb, which did not inhibit growth, coalescence or the formation of specialized cell types, did stimulate a large number of cells to exit and crawl away from the coalescing aggregates. This 3D system, therefore, provides a new level of resolution for studying tumorigenesis *in vitro*.

## Materials and Methods

### Growth and maintenance of cell lines and patient samples

The tumorigenic cell line MDA-MB-435-Br1 (MB-435-Br1) and the more tumorigenic line MDA-MB-435-α6HG6 (MB-435-αHG6) [43,44], were generous gifts from Dr. Suranganie Dharmawardhane of the University of Puerto Rico School of Medicine, San Juan, Puerto Rico. Both of these cell lines are derivatives of MDA-MB-435 [45], originally described as a breast cancer derivative. It was recently discovered that this cell line expressed both breast and melanoma markers [46–48]. Both cell lines were maintained in Dulbecco’s modified Eagle’s medium (DMEM, Life Technologies, Carlsbad, CA) supplemented with 10% (v/v) heat-inactivated fetal bovine serum (FBS, Life Technologies, Carlsbad, CA) and a penicillin/streptomycin mixture (Life Technologies, Carlsbad, CA) [49]. The non-tumorigenic cell line MCF-10A, derived from normal breast epithelial cells [50], was obtained from the American Type Culture Collection (http://www.atcc.org) and maintained in mammary epithelial cell basal medium supplemented with epidermal growth factor, hydrocortisone, insulin (MEGM Bullet Kit, Lonza) and cholera toxin (100 ng/ml, Sigma) [51]. Cells from fresh tumor biopsies and fresh normal tissues were isolated under protocols at Mercy Hospital Systems, Des Moines, IA approved by the Western Institutional Review Board (WIRB), Protocol #20091867 with written informed patient consent to participate in a study entitled “Multi-center Collection of Biospecimens and Associated Clinical Data” and under protocols approved by the University of Iowa Institutional Review Board, IRB ID#200804792, with written informed patient consent to participate in a study entitled “Ocular Skin and Connective Tissue Proliferative Disorders Clinical Data and Tissue Sample Collection Project” through the Melanoma and Sarcoma Tissue Bank Registry (MAST; https://www.icts.uiowa.edu/sites/default/files/VersionApr2013.pdf) at the University of Iowa Carver College of Medicine, project number 028, Dr. Milhem. Tissues were mechanically dissociated and after two to three weeks, a subgroup of growing cells was adapted to DMEM/F12 (Life Technologies, Carlsbad, CA) medium supplemented with 20 ng/ml EGF, 0.5 μg /ml hydrocortisone, 10 μg/ml insulin, 0.1 μg/ml cholera toxin (Sigma-Aldrich, St. Louis, MO), 5% horse serum (Life Technologies, Carlsbad, CA), penicillin/streptomycin and [52,53]. All of the established cell lines and cultures adapted from fresh tumors used in the subsequent studies are described in S1 Table.

### Casting 3D Matrigel cultures

**Fig 1:**
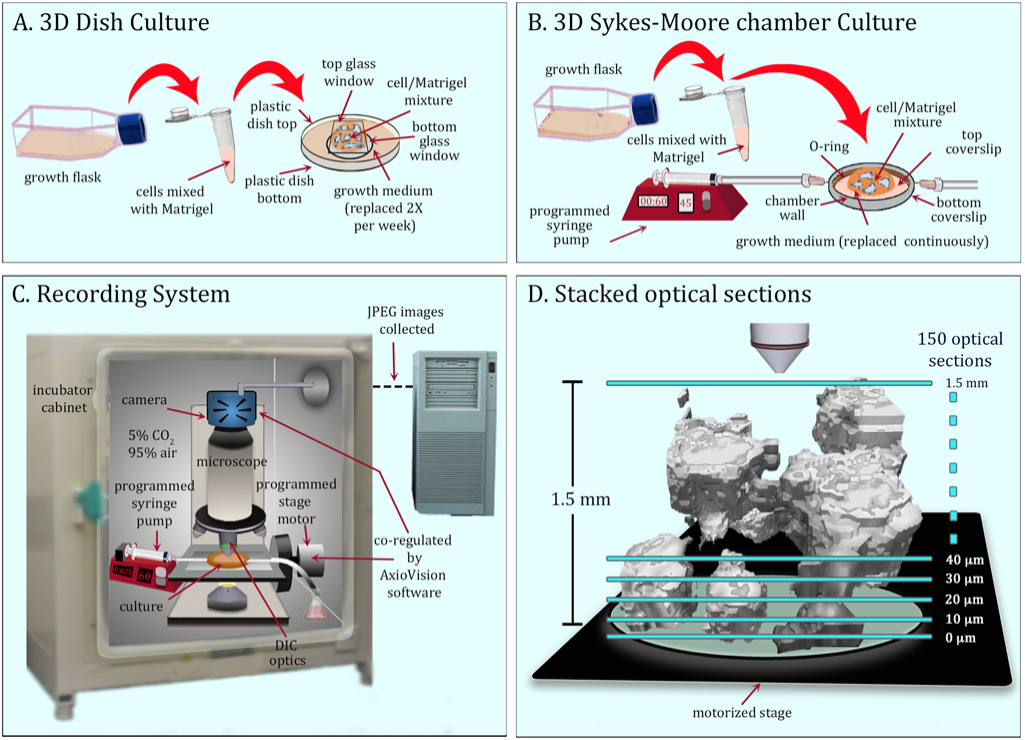
3D culture and image acquisition systems. A. 3D dish culture in which medium is replace twice a week. B. 3D Sykes-Moore chamber in which medium is perfused continuously. C. The recording system in a CO_2_ incubator in which the mechanical stage and camera are coordinated during optical sectioning with AxioVision software. The microscope is fitted with differential interference contrast (DIC) optics. D. Optical sectioning proceeded through 1.5 mm at 10 μm over a 2 minute period resulting in 150 optical sections. For the experiments reported here, this process was repeated every 30 minutes for 17 days or longer.

Two procedures were used for 3D analysis. In the first, 3D preparations were cast in plastic Petri dishes, 65 mm in diameter with a 30 mm glass insert (“bottom glass window”) for DIC imaging (InVitro Scientific, www.invitrosci.com). Five mm was trimmed from the rim of the bottom dish to decrease height in order to eliminate contact between the lid and objective (Fig. 1A). A 25 mm diameter circle was removed from the center of the plastic Petri dish lid and replaced with a glass coverslip (“top glass window”) for DIC imaging (Fig. 1A). The bottom glass window was pre-coated with 100 μl of Matrigel matrix (Becton Dickinson Bioscience, Franklin Lakes, NJ), which contains laminin, collagen IV, proteoglycan, entactin and growth factors, and incubated for 20 minutes at 37°C. Cells were harvested from growth flasks prior to 80% confluency by trypsinization, diluted to 5x10^5^ cells per ml, chilled on ice, and a 250 μl aliquot mixed with 500 μl of liquefied Matrigel at 4°C. The 750 μl cell-Matrigel mixture was cast on the pre-coated bottom glass window (Fig. 1A) and the preparation incubated for 30 minutes to allow gelation. Medium was then added to the dish and gently replaced at three day intervals over the course of the experiment. In a second procedure, the cell/Matrigel mixture was cast on the bottom glass wall of a Sykes-Moore perfusion chamber, and fresh medium continuously perfused through the chamber, using a syringe pump (NE-1000, New Era Pump Systems, Wantagh, NY) attached to the intake port [54]. Medium was removed through an exit tube connected to the outlet port at the opposite side of the metal wall of the perfusion chamber (Fig. 1B). Repeat experiments revealed no differences in the results obtained using the alternative methods.

### The cabinet

Both types of culture (Fig. 1A and B) were maintained in an incubator at 37°C in 5% CO_2_ (95% air) (Fig. 1C). A culture was positioned on the stage of a Zeiss Axioplan 2 upright microscope equipped with differential interference contrast (DIC) optics, a motor-driven stage, and a Zeiss AxioCam MRc5 IEEE 1394 color CCD camera. For cultures in Sykes-Moore chambers (Fig. 1B), the programmed syringe pump was positioned in the CO_2_ incubator (Fig. 1C).

### Collecting optical sections

Imaging was performed through a 20X long working distance DIC objective with a numerical aperture of 0.4. The z-axis movement of the stage during optical sectioning and the image acquisition cycle of the camera were synchronized by Zeiss AxioVision software. A total of 150 optical sections were acquired in the Z axis at 10 μm increments in 45 seconds (Fig. 1D). The region therefore was 1.5 mm high and 446 μm wide by 335 μm length. This procedure was repeated every 30 minutes for 17 days or longer for the studies reported here. The system has the capacity to repeat optical sectioning every 30 seconds, if desired, to collect more optical sections through a much greater depth, to collect optical sections at Z-axis increments as small as 0.5 μm, and to employ higher magnification. Under the regime used here, we obtained 816 3D reconstructions in 17 days. This amounted to a total of 122,400 optical sections per preparation per period of analysis. The optical sections were delivered to the hard drive of the controlling computer as grayscale JPEG images with moderate compression to preserve the grayscale range, and then transcribed into a new file format native to the software program J3D-DIAS 4.1. Details of file collection are presented in S1 Methods. Select DIC sections from a single z-series of aggregates in a 3D area of a Matrigel preparation are presented in S1A Fig. and select DIC optical sections of a single cell, at higher magnification, in S1E Fig.

### Edge detection

An automatic object detection algorithm, “complexity-based bitmap object detection” (C-BBOD), which we developed to automatically identify the in-focus aggregate images in each optical section, is described in S1 Methods. A manual tracing method applied to some single cells and filopodial extensions is also described in S1 Methods. The C-BBOD algorithm distinguished the in-focus region of aggregates in each optical section (S1A Fig.), stored this information as data “bits” and rendered these bits as pixels (see purple regions in S1B Fig.). The C-BBOD images of aggregates within the 150 optical sections were stacked for reconstruction (see S1C Fig.). For manual outlining, J3D-DIAS 4.1 employed portions of the original 2D-DIAS program [27,28,30,31,55] as described in S1 Methods. In brief, the in-focus edge of a cell in each optical section (S1E Fig.) was discerned by the user and outlined, using the mouse to generate a continuous perimeter. Points in the outline at selected distances were then automatically connected with straight lines and smoothed with a beta spline algorithm (S1F Fig.). The beta spline models were stacked at each time point (S1G Fig.). Filopodia were manually identified and drawn as lines (see green lines in S1F Fig.).

### Generating encapsulated 3D images

**Fig 2:**
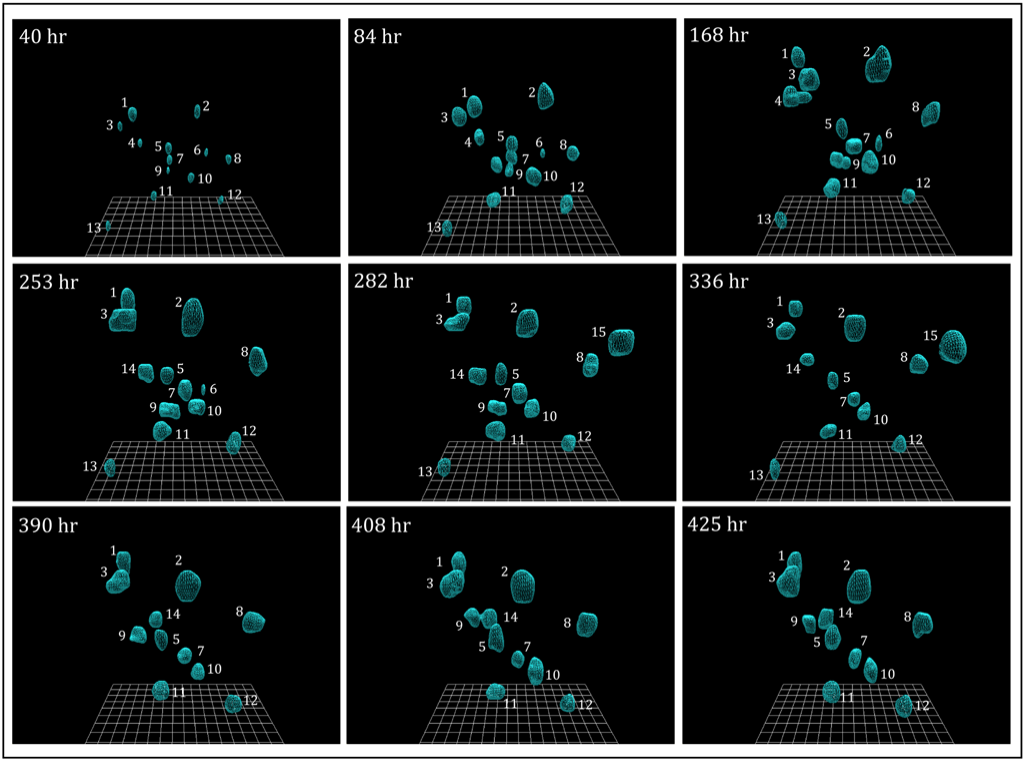
3D reconstructions of preparations of the non-tumorigenic cell line MCF-10A. MCF-10A cells multiplied to form aggregates, but the aggregates remained, for the most part, in place and did not coalesce. Initially, 13 aggregates formed, three exited permanently, one transiently and two entered the field of analysis. The changes occurred at the edge of the visual field. Reconstructions were generated using the automatic tracing (C-BBOD) and smoothing programs described in S1 Methods.

**Fig 3:**
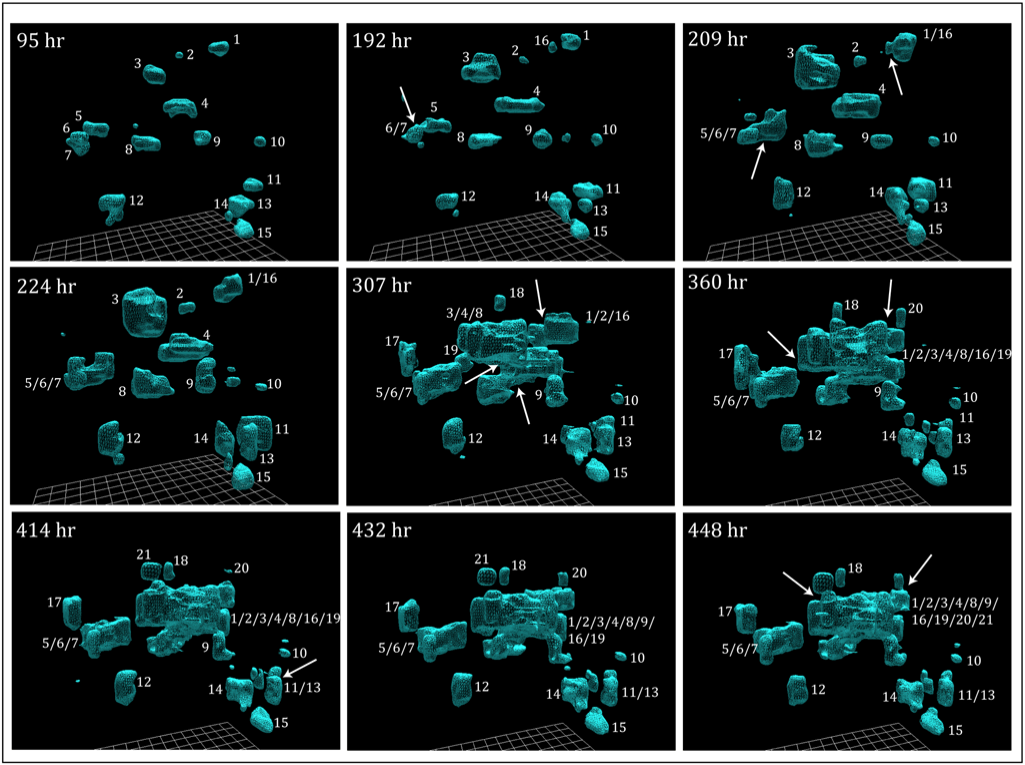
3D reconstructions of preparations of the tumorigenic cell line MB-435-Br1. MB-435-Br1 cells multiplied to form independent aggregates through 140 hours, but these aggregates then began to coalesce. Initially, at 95 hours, 15 aggregates had formed, but the number rose to 21 as aggregates entered the 3D area of analysis at its edge. Reconstructions were generated using the automated tracing (C-BBOD) and smoothing programs described in S1 Methods. Arrows indicate examples of coalescence.

In a z-axis series of either autotraced or manually traced images, stacked traces were separated by 5 or 10 μm, the distance between the collected optical sections. These traces were connected in the z axis to render 3D reconstructions by the “adaptive skeleton climbing” technique [56]. This method is described in S1 Methods. The surface of reconstructed aggregates or cells could then be viewed in 3D as solid, shadowed, nontransparent objects, such as those in S1D and S1H Fig., or as transparent caged aggregate images, such as the aggregate images in Figs. 2 and 3.

### Quantitating 3D velocity and contour parameters

“The percent original aggregate number” took into account aggregates at the edge entering and exiting the area of view. The position of the 3D “centroid”, the center of the 3D object, determined from the averaged x, y, and z coordinates of the pixels in an aggregate or cell reconstruction, was used to calculate “mean speed”. Surface complexity and volume were calculated from the 3D faceted mathematical models of the reconstructed aggregates. All parameters were computed at 30 min intervals and are defined in S2 Methods.

### F-actin and DNA staining of mature aggregates

MB-435-Br1 aggregates were cultured and stained using the protocol developed by Lee et al [57] with minor modifications. Thirty day dish cultures, described in a previous section, were washed in phosphate buffer solution (PBS) and fixed for 15 minutes at room temperature in 2% paraformaldehyde. After three washes in PBS, the preparation was permeabilized for 5 minutes in 0.2% Triton X-100, washed in PBS, blocked in 1% BSA for 20 minutes and then washed again. For actin and DNA staining, respectively, 10 μl of a stock solution of Alexa Fluor 488 conjugated phalloidin (Life Technologies, Carlsbad, CA) was diluted into 200 μl PBS pH 7.4 containing Sytox Orange (Life Technologies, Carlsbad, CA) diluted 1:2000. The sample was incubated for 20 minutes followed by three washes in PBS. Images were acquired with a Nikon TE2000 inverted fluorescence microscope (Nikon Instruments, Melville, NY) connected to a Bio-Rad RadianceMP 2100 laser scanning confocal microscope.

### Treatment of 3D cultures with monoclonal antibodies (mAbs)

Concentrated supernatants of mAb AIIB2 (ß-1 integrin) and mAb P1B5 (α-3 integrin) without the anti-microbial agent ProClin 150 were obtained from the Developmental Studies Hybridoma Bank (DSHB; Department of Biology, University of Iowa, Iowa City, IA 52242; http://dshb.biology.uiowa.edu). The mAbs were either incorporated into the cell-Matrigel culture, prepared as described above (Fig. 1A), or introduced and maintained by perfusion through cultures in the Sykes-Moore Chamber (Fig. 1B) at a concentration of 100 μg/ml in both cases. The two methods yielded identical results.

## Results

### The behavior of no tumorigenic cancer cell aggregates

We first analyzed the aggregation behavior of cells of a non-tumorigenic cell line, MCF-10A [50] (S1 Table). Reconstructions in one area of analysis are provided over a period of 17 days in Fig. 2. Over 90% of cells dispersed in this area of the 3D Matrigel multiplied for approximately seven days without noticeable changes in position. The increases in size and the stability of the relative positions are evident in comparisons of the reconstructions at 40, 84 and 168 hours (Fig. 2). Aggregates remained relatively fixed positionally in the gel for 11 subsequent days (Fig. 2). Non-uniform shrinkage of the gel brought a few cells at the outer edge of the 3D area of analysis in and out of view. This was evident between 168 and 282 hours by the loss of aggregates 4 and 6, the appearance of aggregate 14 and the transient appearance of aggregate 15 (Fig. 2). None of the aggregates formed by MCF-10A cells coalesced over the 425 hours of analysis. Qualitative experiments performed beyond 17 days revealed no subsequent coalescence. The absence of coalescence was also verified by examining reconstructions at intervals shorter than 30 minutes (data not shown) and by rotating images (S2A Fig.). Coalescence did not occur even when aggregates, such as aggregates 7 and 10, came in close contact at 253 hours (S2A Fig.). An analysis of the inter-aggregate space revealed no individual cells exiting the aggregates and no cells at the aggregate surface probing the inter-aggregate spaces (S3A Fig.). Repeat analyses of preparations of MCF-10A cells yielded the same scenario described above (Fig. 2; S1 Movie) and in S2A Fig., as did repeat experiments with preparations of MARI 027 cells, obtained from normal lung tissue (S1 Table).

### The behavior of tumorigenic cell aggregates

The same methods were then used to reconstruct cells of the tumorigenic cell line MB-435-Br1 (S1 Table). Select reconstructions in the area of analysis are provided over a period of 19 days (Fig. 3). Over the first 95 hours, seeded cells multiplied in place to form aggregates, in a manner similar to control cells (Fig. 2). However, in contrast to the aggregates formed by MCF-10A and MARI 027 cells, those formed by MB-435-Br1 varied in shape dramatically from that of a sphere, and a review of select reconstructions prior to 90 hours revealed constantly changing shapes. These shape changes continued and were evident in temporal comparisons of the reconstructions of aggregates, for example aggregates 3, 4, 8 and 11, at 95 and 192 hours (Fig. 3).

By 192 hours, closely associated MB-435-Br1 cell aggregates began to coalesce (white arrow, Fig. 3, 192 hour). Analyses of individual DIC sections, 3D reconstructions at short time intervals (data not shown) and rotation of 3D reconstructions (S2B Fig.) verified that coalescence began after 140 hours in the 3D MB-435-Br1 preparation in Fig. 3. After each coalescence, the resulting fused aggregates have been coded with a slash, providing the reader with a history of coalescence. Therefore, when aggregates 6 and 7 coalesced between 95 and 192 hours, the product was denoted as aggregate 6/7 (Fig. 3) and when aggregates 1 and 16 coalesced between 192 and 209 hours, the product was denoted as aggregate 1/16 (Fig. 3, arrows). When the coalesced aggregate 6/7 in turn coalesced with aggregate 5 between 192 and 209 hours, it was referred to as aggregate 5/6/7 (Fig. 3). Over the period of analysis, five aggregates originally outside the field of view (17 to 21), entered it. Over the entire period of analysis (448 hours), 12 aggregates coalesced, reducing the total number of independent aggregates in the field of analysis, including original aggregates and those entering the field, from 19 to 9. Coalescence was verified by rotation (S2B Fig.). By 448 hours, ten of the original aggregates (1, 2, 3, 4, 8, 9, 16, 19, 20, 21) had fused to form one large aggregate (Fig. 3, 448 hours). Later reconstructions in repeat experiments revealed that aggregates continued to coalesce.

In contrast to non-tumorigenic cell preparations, DIC images of the inter-aggregative spaces of tumorigenic cell preparations revealed single cells extending from the aggregate surfaces, and single cells exiting aggregates and entering the inter-aggregate spaces (starred cells in S3B Fig.). These single cell behaviors were verified by examining DIC images in optical sections, at the same height in the gel, at 30 minute time intervals (data not shown). The coalescence of aggregates (Fig. 3) and the entry of cells into inter-aggregate spaces (S3B Fig.) described for preparations of tumorigenic MB-435-Br1 cells, were observed in repeat experiments of this cell line, and in preparations of the cell lines MB-435-α6HG6, LN18 and U87, and the cell preparations derived from fresh tumor cell preparations MARI 011, MARI 023, MARI 028 and MARI M2 (S1 Table; S2 Movie).

### Quantifying aggregate behavior

Because every aggregate in a monitored area of the Matrigel was converted to a 3D mathematical model in the process of reconstruction (S1 Methods), 3D contour and motility parameters could be readily quantified at 30 minute time intervals through the period of analysis [27,28]. Three of these parameters as well as percent original aggregates are plotted in S4 Fig. for the field of analysis of the representative non-tumorigenic cell line MCF-10A (green plots in S4 Fig.) and the representative tumorigenic cell line MB-435-Br1 (blue plots in S4 Fig.). The four parameters plotted were the “percent original aggregate number”, “mean aggregate volume” (μm^3^), “mean aggregate surface complexity” and “mean aggregate 3D speed” (μm/hr). Formulations of the four parameters are provided in S2 Methods. For MCF-10A preparations, the percent original aggregate number remained constant for 340 hours (S4A Fig.). Mean volume increased during the first 80 hours, remained relatively constant for the next 150 hours, decreased between 240 and 270 hours and then remained constant between 270 and 340 hours (S4B Fig.). Mean surface complexity remained relatively constant between 0 and 225 hours decreased slightly between 225 and 280 hours, then remained relatively constant (S4C Fig.). Finally, mean speed of MCF-10A aggregates remained relatively constant throughout 340 hours (S4D Fig.). The small changes in volume, mean surface complexity and mean speed (S4B, C and D Fig., respectively), appeared to be due to compacting of the aggregates between 240 and 280 hours.

In marked contrast, dramatic changes occurred in MB-435-Br1 preparations after 170 hours of incubation. As the number of aggregates continuously decreased due to coalescence beginning at approximately 170 hours (S4A Fig.), the mean volume of aggregates increased at a relatively constant rate (S4B Fig.). Mean surface complexity increased continuously between 90 and 340 hours (S4C Fig.), reflecting the variation in aggregate contours and the complex shapes resulting from coalescence, such as that of aggregate 1/2/3/4/8/9/16/19/20/21 at 448 hours (Fig. 3). The mean speed of aggregate translocation nearly doubled between 170 and 210 hours, then remained high through 340 hours (S4D Fig.).

### Special cell types mediate coalescence

Two observations led us to consider the possibility that specialized cells played a role in mediating aggregate coalescence in tumorigenic cell lines or fresh tumor preparations. First, it was observed in all coalescing preparations that smaller aggregates were drawn to larger ones in a nonrandom fashion. Coalescence, hence, did not appear to be due to random collisions. Second, it was observed, as noted, that cells entered inter-aggregative spaces only in tumorigenic cell preparations (compare S3A Fig. and S3B Fig.). We therefore reconstructed preparations using automatic C-BBOD tracing software for aggregates and manual tracing software for cells of interest that exited aggregates into an inter-aggregate space and cells that projected from aggregate surfaces (S1 Methods). Two types of single cell behavior that facilitated coalescence were identified, one involving two cell types and the other just one of the two.

**Fig 4:**
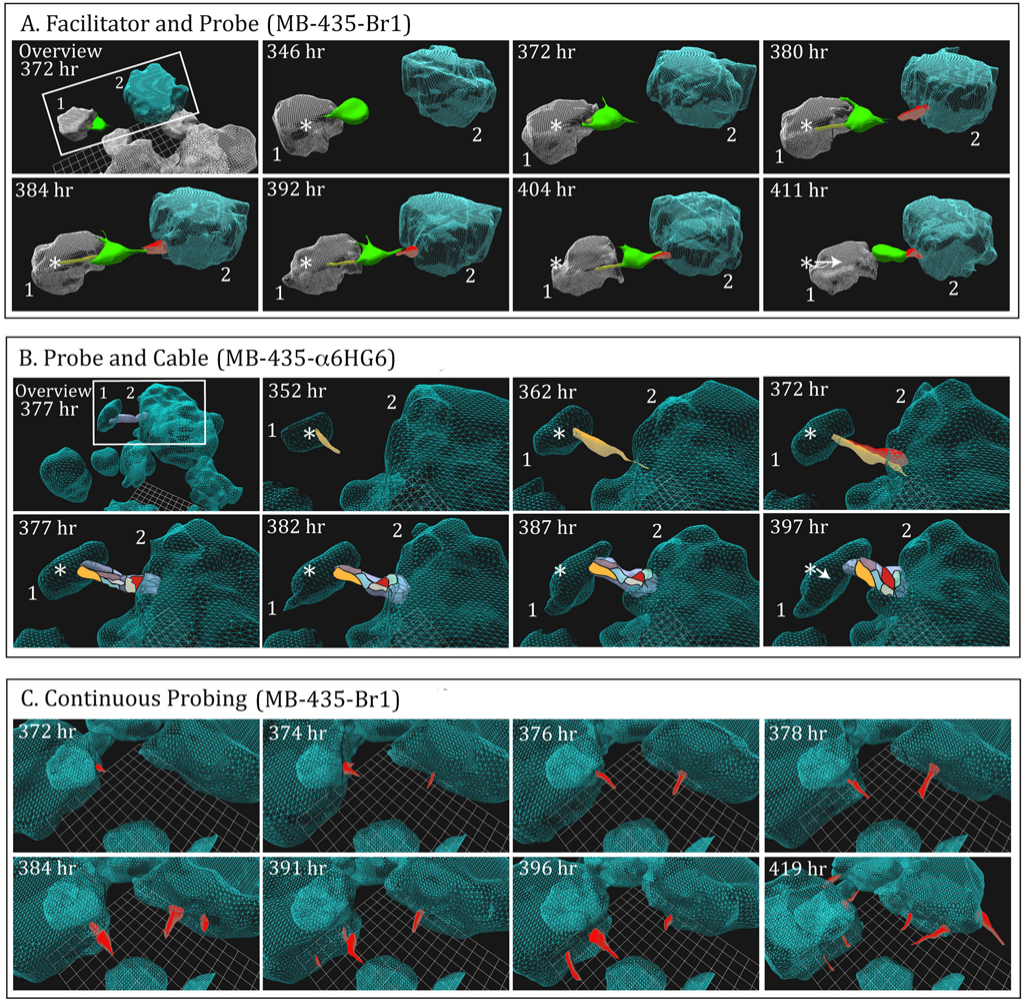
Specialized cellular interactions facilitate coalescence after 140 hours in tumorigenic cell lines. A. An example of the facilitation of coalescence of two aggregates (blue and gray) through interactions of a multinucleated “facilitator” cell (green) and “probe” cell (red) in a preparation of MB-435-Br1 cells. The overview in the first panel is a lower magnification image that included the boxed-in area of analysis at 372 hours. The distance of translocation by the small aggregate (gray) towards the large aggregate (blue) is indicated by the arrow in the panel for 411 hour, originating at the position of the centroid (star) of the smaller (gray) aggregate at 346 hours. The filopodia extending from the facilitator cell around the aggregate of origin are color-coded yellow. Note the contact of a protrusion from the facilitator and probe cells at 392 hours and contraction at 411 hours. B. An example of probe cells alone facilitating coalescence by forming a cable which contracts. The overview panel, arrow and star are explained in the legend to Fig. 4. Probe cells are color-coded yellow, red, gray, brown, blue and purple. Note formation of a putative filopodium contacting the large aggregate at 362 hours, extension of a probe cell at the point of filopod contact, contact of the first two probe cells at 372 hours, cable formation at 377 hours and cable contraction by 397 hours. C. Coalescing aggregates in the tumorigenic cell line MB-435-Br1 continually extend multiple probe cells in various directions. In panels A and B, only the cells facilitating coalescence were reconstructed, but in panel C, multiple, but not all, probe cells were reconstructed to exemplify the randomness of probing. In all reconstructions (A, B, and C), the aggregates were generated by autotracing (C-BBOD) and the cells by manual tracing, as described in S1 Methods.

**First scenario.** An example of the first scenario is presented for a MB-435-Br1 preparation in Fig. 4A. The area of analysis, an inter-aggregate space, is windowed in a low magnification 3D view of the field, in the first panel (“Overview 372 hr”, Fig. 4A). In this scenario, a large cell (color-coded green), containing five nuclei determined from DIC optical sections, exited aggregate 1 into the space between it and aggregate 2 (Fig. 4A, 346 hr). After exiting, it remained attached to aggregate 1, extending filopodia over that aggregate’s surface, as if anchoring itself to the aggregate (Fig. 4A, 346 to 380 hr). At 380 hours, a cell (color-coded red) protruded from the surface of the larger, neighboring aggregate towards an opposing filopodium emanating from the large (green) cell, and extended directly towards the former cell (Fig. 4A). The extensions from the large cell and the cell protruding from the large aggregate were aligned perfectly, suggesting that the filopodia extending from the tip of large cell (green) had made contact with the protruding cell (red) (Fig. 4A, 380 hr). Imaging the full length of the distal portion of these filopodia was beyond the sensitivity of our imaging procedure as performed here. The multinuclear large cell that had exited from aggregate 1 and then anchored to it, we have named the “facilitator” cell, and the cell protruding from the large aggregate surface, we have named “probe” cell. They visibly contacted one another end-to-end by 384 hours, extended along each other and then contracted (Fig. 4, 384 hours to 411 hours), with enough force to pull the small aggregate towards the larger one, as is evident by the translocation arrow in the panel for 411 hours (Fig. 4A). This arrow reflects the distance traveled by the centroid (center of mass) of the smaller aggregate between 384 and 411 hours.

Facilitators and probes were observed not only in preparations of MB-435-Br1 (S3 Movie), but also in MB-435-α6HG6 preparations, a cell line derived from a glioblastoma (U87), a cell preparation derived from a fresh biopsy of a kidney tumor (MARI 023 paraganglioma) and in MARI M2, derived from an aggressive melanoma (S1 Table).

**Second scenario.** The alternative scenario is demonstrated in a preparation of MB-435-α6HG6 cells (S1 Table). For this behavior, the area of analysis, which included an inter-aggregate space, was windowed in the low magnification 3D image of the field (“Overview 377 hr”, Fig. 4B). In this scenario, a probe (yellow) from a smaller aggregate protruded towards a larger aggregate at 352 hours, and extended a filopodium that contacted the surface of the large aggregate by 362 hours (Fig. 4B). This caused protrusion of a probe cell (red) from the large aggregate at the point of contact by 372 hours. This latter probe cell extended along the probe cell of the smaller aggregate (Fig. 4B, 372 hours). Additional probe cells (blue, purple, gray) then extended from the larger aggregate, forming a thick cabled bridge between the two aggregates. This cabled bridge thickened and contracted between 372 and 397 hours, moving the smaller aggregate towards the larger one, as evidenced by the translocation arrow (Fig. 4B, 397 hours).

Probes were observed protruding from the aggregates of every tumorigenic cell line and in all fresh preparations from tumors examined to date (S1 Table), as noted. Probes were not observed in the two control preparations, MCF-10A and MARI 027, but were observed in the non-coalescing cell line MCF-7 (S1 Table).

### Probe cells extend from aggregates in random directions

Although we limited our reconstructions to the probe cells facilitating coalescence in the representative examples of the two scenarios, additional probe cells continually extended from aggregates without an opposing response by a surface cell of a neighboring aggregate and into regions with no opposing aggregates (Fig. 4C). These randomly positioned probes continually expanded and contracted. The filopodia formed by these probe cells were not reconstructed.

### The “dervish”

**Fig 5:**
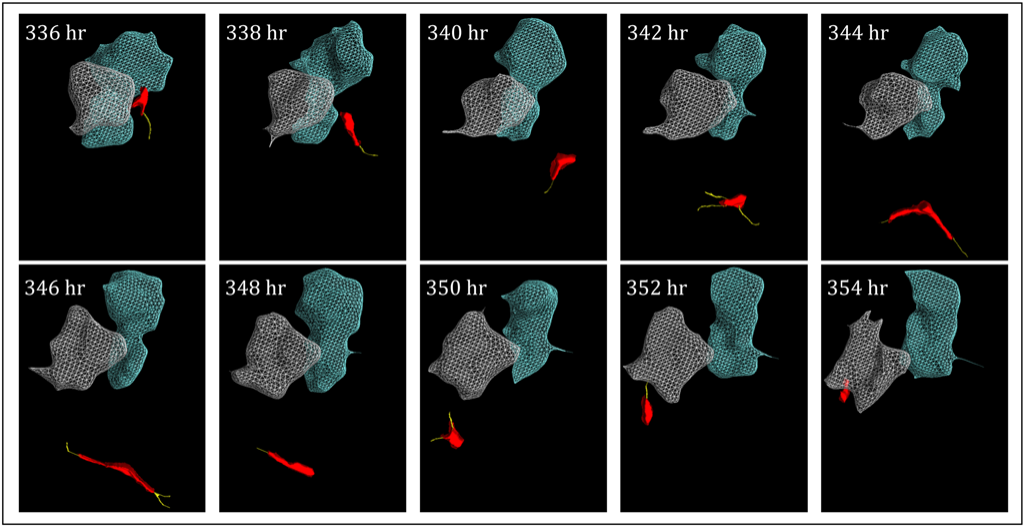
A “dervish” cell (red) released by an MB-435-Br1 aggregate after 140 hours, moves in a swirling manner along an erratic path through the Matrigel, extending pseudopodia (yellow) from multiple projections. Although dervish cells frequently contact aggregates, they do not remain attached to them. Aggregates are color-coded gray and blue. Aggregate reconstructions were generated by autotracing and the cell reconstructions by manual tracing, as described in S1 Methods.

In 3D preparations each of four tumorigenic cell lines and in 3D preparations of two lines from fresh tumors, we observed, mononucleate cells the size of probe cells that exited aggregates, but did not play a part in coalescence (S1 Table). The behavior of this cell type is demonstrated in a MB-435-Br1 cell preparation reconstructed in Fig. 5, in which we present reconstructions of two aggregates (gray and blue) automatically detected by the C-BBOD algorithm and the unique cell type (red) generated in manual tracing, between 336 and 354 hours of analysis. The cell moved through the Matrigel along a random, swirling path, hence the name “dervish”, changing speed and reaching extremely high velocities (S5A Fig. and S5B Fig.; S4 Movie). When this cell contacted an aggregate, as in panels 336 and 338 hours of Fig. 5, it did not integrate back into the aggregate, but moved rapidly away. Dervishes attained velocities greater than 25 μm per hour (S5B Fig.) along their random paths (S5A Fig.). In addition to moving rapidly through Matrigel, dervishes were also observed on rare occasions to enter small aggregates on one side and several hours later exit the aggregate on the other side (data not shown). Hence, dervishes have the remarkable characteristics of moving through Matrigel at extraordinarily high speeds, lack of adhesion to aggregates and the capacity to pass through aggregates.

### Mature aggregates develop into hollow spheres that can collapse

**Fig 6:**
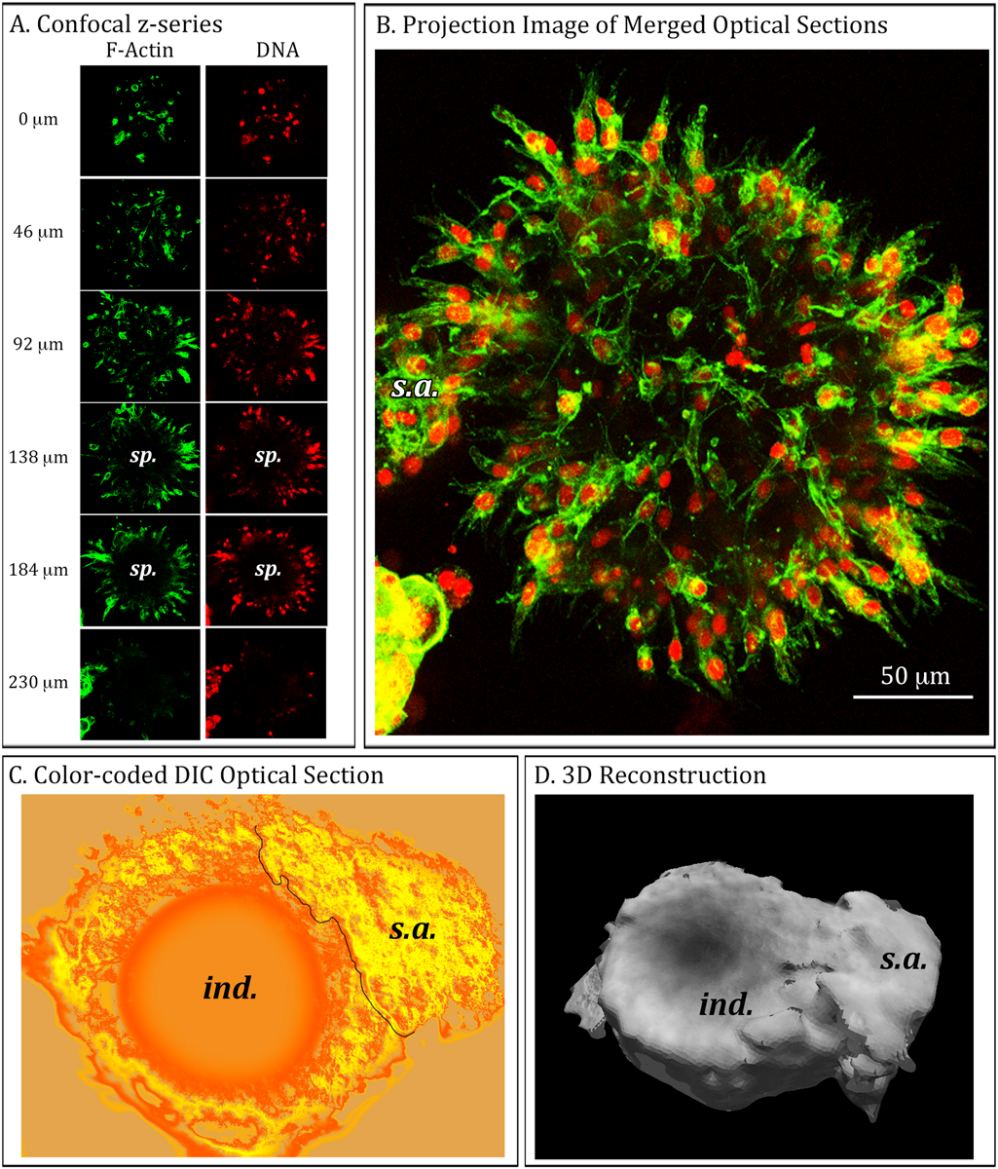
Optical sections and 3D reconstructions of both stained and live MB-435-Br1 aggregates demonstrate that by 30 days in 3D culture, large aggregates assume a sphere shape which, in many cases, collapses. These spheres continue to undergo coalescence with neighboring solid aggregates. A. A representative z-series at 46 μm intervals acquired by confocal microscopy (left side of panel) through 230 μm of a representative aggregate that formed after 30 days of culture in 3D Matrigel. The preparation was fixed and then stained for F-actin (green) and DNA (red). B. A projection image of the merged F-actin and DNA z-series acquired by confocal microscopy. Note the presence of a small secondary solid aggregate (s.a.) attached to the spherical aggregate. C. A color-coded optical section of a live, representative aggregate acquired by DIC microscopy highlights a cup-shape with a round indentation (ind.) caused presumably by the collapse of the sphere. The line in panel C demarcates the boundary between the concave aggregate and a merging, small solid aggregate (s.a.). D. J3D-DIAS 4.1 3D reconstruction of the entire z-series of optical sections using C-BBOD with secondary aggregate (s.a.) noted.

In the preceding studies we analyzed aggregates coalescence through approximately 17 days of culture. In most 17 day preparations, aggregates were amorphously shaped, with no symmetric architecture (Fig. 3). However, if MB-435-Br1 preparations were incubated in Matrigel for 30 days, close to 50% developed into hollow spheres, some of which collapsed. In Fig. 6A and B, we present examples of such mature aggregates using fluorescent microscopy. The mature aggregate was stained with Alexa Fluor 488-conjugated phalloidin to visualize actin distribution, and with DAPI to visualize nuclei (DNA). When the green actin and red DNA staining overlap, the combined color is yellow (Fig. 6B). Representative confocal sections between 0 and 230 μm through the aggregate revealed that the distribution of both actin and nuclei, and hence cells, were localized at the perimeter of a sphere and that the larger core space (sp) was devoid of cells (Fig. 6A). Projection images of the overlaid actin stained and DNA-stained collections of optical sections revealed a hollow sphere in which cells exhibited orientation (Fig. 6B). The cells were elongate and their long axis was oriented perpendicular to the sphere edge. In Fig. 6C, we present an example of a pseudo-colored DIC optical section through a collapsed sphere revealing a donut-shaped image with an indentation, (“cav”), presumably where one side of the sphere had collapsed up against the far side (Fig. 6C). The collapse is evident in the C-BBOD reconstruction of this aggregate (Fig. 6D). In both the stained confocal projection (Fig. 6B) and the C-BBOD reconstructions (Fig. 6C and D), an amorphously shaped, small solid aggregate (s.a.), had contacted and adhered to the large, mature sphere. In the DIC optical section of this second example (Fig. 6C), a clear demarcation is apparent at the contact, suggesting cohesion had occurred, but cellular integration was not yet complete. Interestingly, the small aggregate (s.a.) adhering to the fluorescently-stained sphere in Fig. 6B also had not yet integrated.

### Assessing the effects of monoclonal antibodies

The usefulness of 3D models for assessing the effects of anti-cancer drugs and monoclonal antibodies (mAbs) has been proposed and explained in previous studies [13,15,26]. The discovery of coalescence, the identification of facilitating cell types, the capacity to generate true 4D reconstruction and quantification of contours and motility parameters, using J3D-DIAS 4.1, should enhance the usefulness of such 3D analyses of drug effects. To verify such usefulness, we analyzed the effects on MB-435-Br1 preparations of two monoclonal antibodies. The monoclonal antibodies were either mixed with the Matrigel (Fig. 1A) or perfused throughout incubation by adding them to the medium pumped through cultures in the Sykes-Moore Chamber (Fig. 1B). The first drug tested, AIIB2, is a mAb that targets ß-1 integrin. This mAb was shown [13] to cause reversion of the amorphous 3D phenotype of aggregates formed by the human breast cancer cell line T4–2 to the sphere formed by non-tumorigenic cells. The second, PIB5, targets α-3 integrin, which interacts with ß-1 integrin [58] and tetraspanin in the regulation of tumor cell invasion [59].

**Fig 7:**
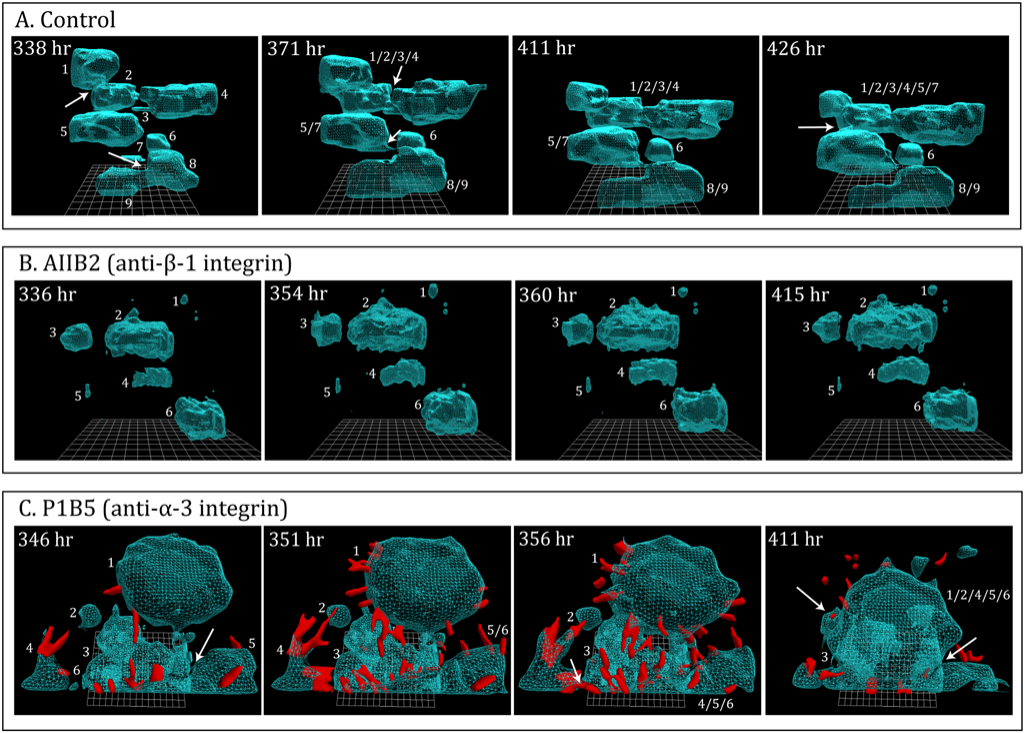
A demonstration that this system can be used to assess the effects of monoclonal antibodies (mAbs) upon the process of aggregate formation and coalescence. Preparations of MB-435- α6HG6 cells were continuously infused with either of two anti-integrin mAbs. A. Untreated control preparation of aggregates (blue) undergoing coalescence between 338 and 426 hours. In this period, eight of the nine aggregates coalesced. B. A preparation of aggregates (blue) treated with the anti-ß-1 integrin mAb, AIIB2, underwent growth but did not coalesce. C. A preparation of aggregates (blue) treated with the anti-α-3 integrin mAB, P1B5, underwent accelerated coalescence, and exhibited a massive increase in the formation of probe cells (red), many of which exited the aggregates.

The anti-ß-1 integrin mAb AIIB2 had no effect in the growth of aggregates, which attained roughly the same average volumes (Fig. 7B) as untreated control cultures (Fig. 7A). However, in contrast to untreated control cultures (Fig. 7A), coalescence was blocked in treated cultures (Fig. 7B). An analysis of DIC optical sections revealed the absence of facilitator and probe cells throughout the time of incubation, in direct contrast to maturing control preparations (data not shown). Preparations perfused with PIB5 underwent growth and increased coalescence (Fig. 7C). However, DIC images revealed not only an increased frequency of probes extending from PIB5-treated aggregates, but also the massive exit of probe cells from the coalescing aggregates (Fig. 7C).

## Discussion

We have developed a computer-assisted system for reconstructing and motion-analyzing, in 4D, cancer cells multiplying and undergoing aggregate coalescence in transparent Matrigel. Because the system relies on DIC optics rather than confocal microscopy, there is no photoxicity; therefore, reconstructions can be performed at intervals as short as every 30 seconds for unlimited periods of time. Because aggregates can be automatically reconstructed using complexity-based bitmap object detection (C-BBOD), the system has the capacity to produce 21,600 reconstructions at 2 minute time intervals for periods of 30 days or more. Presently single cells are traced manually. The user can select single cell behaviors for the more time-consuming task of manual tracing. It should be noted that although the 3D-DIAS program was established over two decades ago for single cells and modified for the 3D reconstruction of embryos in 2002 [37], it took roughly three years to adapt those earlier technologies to perform the reconstructions presented here.

### Coalescence

Comparisons of reconstructions of non-tumorigenic and tumorigenic cell preparations revealed a number of differences in behavior that suggested the latter underwent a developmental program that led to aggregate coalescence involving the formation of specialized cell behaviors and terminal aggregate morphogenesis. Both non-tumorigenic and tumorigenic cells seeded in Matrigel multiplied into independent islands of cells during the first 100 to 140 hours of incubation. The only difference was in the shape of the aggregates. Tumorigenic cell aggregates underwent continuous shape distortion, suggesting behavioral activity, whereas non-tumorigenic cell aggregates remained relatively round, suggesting inactivity. Between 100 and 140 hours of incubation, aggregates in preparations of the tumorigenic cell line began to coalesce. Coalescence between neighboring but independent aggregates occurred in an apparently directed fashion, suggesting it was not due to random collision. Coalescence continued until the majority of aggregates in a 3D area of analysis were incorporated into a single large aggregate. Although the process of coalescence has not to our knowledge been specifically described previously in seeded Matrigel preparations, it has been suggested in a model developed by Sloane and coworkers [60] in which cancer cells are dispersed on the top of a two layer gel, the top layer containing reconstructed basement membrane, DQ-collagen and fibroblasts.

### The facilitation of coalescence

Concomitant with coalescence in preparations of all but one of the tumorigenic cell lines and in preparations we generated from fresh tumors, cells protruded from the surface of the aggregates and retracted in an apparently random fashion. Because of this probing behavior and their subsequent roles in facilitating coalescence, we have labeled these cells “probe cells”. They are visible in DIC images and must be manually traced, since the smoothing algorithms of C-BBOD and subsequent reconstruction did not preserve their morphology. Probes in many cases extended long filopodia that contacted other filopodia or cells at the surface of a neighboring aggregate. This interaction induced directed extension of a cell protrusion of the opposing cells of two neighboring aggregates. When the cell bodies of the two opposing probes made contact end to end, they wrapped around one another to form an initial cable. Additional cell protrusions adjacent to the original cabled probes extended out along the initial cable to form a thick cable, which then contracted, moving the smaller aggregate towards the larger one. Probe cells did not appear in aggregates of non-tumorigenic cell lines, which also did not undergo coalescence. Probe cells did appear in aggregates of a very weakly tumorigenic cell line, MCF-7, which did not coalesce. All nine tumorigenic cell lines and fresh tumor cell preparations that underwent coalescence formed probes (S1 Table). The origins of these cell preparations included melanoma, breast tumor, glioblastoma, omentum, kidney, parganglioma and lung tumor (S1 Table). In one line from a highly aggressive melanoma, MARI M2, the speed of coalescence was so high that the role of interstitial cells was difficult to assess. This preparation is now being studied at far shorter intervals between reconstructions.

A second cell type was observed that was large and multinucleated. These cells left the aggregate, but then anchored themselves to the aggregates through the extension of adhesive processes. These large anchored cells protruded into the inter-aggregate spaces, extending long filopodia, which contacted probe cells on a neighboring aggregate. The large cells and the probe cells they contacted underwent contraction in unison, moving the smaller aggregates towards the larger ones. Because of their role in facilitating coalescence, we have labeled these larger cells “facilitator cells”. Facilitators were observed in five of the ten cell lines or fresh tumor preparations that underwent coalescence.

The facilitator cell is large, multinucleated, multi-processed, and appears to be functionally specialized. However, probe cells may simply represent cells that are serendipitously located at the outer surface of solid aggregates and may exhibit behaviors inherent in all cells in the aggregate. To resolve this question, biomarkers are being sought that may selectively distinguish the probe and facilitator cell types. However, it was clear in multiple preparations and cell lines that the capacity to form cells with the behaviors of probes and facilitators was not attained until after 100 hours, suggesting that the behaviors, if not the formation of specialized cell types, may actually represent a stage programmed into the development of cancer cell aggregates formed in Matrigel. The role of filopodia was clear and consistent with their role in sensing and redirecting translocation in other cell types [36,61,62].

### The dervish

Dervishes were observed in preparations of six of the nine cell preparations that underwent coalescence, but not in preparations that did not undergo coalescence (S1 Table). These cells moved at remarkably high speeds and presumably had to digest the Matrigel matrix in order to do so. Moreover, dervishes did not adhere to cell aggregates, as was the case for cells within the aggregate, probes and facilitators. We observed in several preparations dervishes entering aggregates and a few hours later exiting them. Their unique loss of constraints suggested that if they were formed in natural tumors, they would likely be highly migratory.

### The effect of mAbs on aggregation

To prove the usefulness of the system for assessing the effects of known and potential anti-tumor drugs, we tested two mAbs. AIIB2, an anti-ß-1 integrin mAb, had already been shown by Bissell and coworkers [13] to disrupt the architecture of breast-derived cancer cell aggregates formed in 3D, in essence causing reversion to the phenotype of the non-tumorigenic cell line from which they were derived. P1B5, an anti-α-3 integrin mAb was shown to have no effect on colony formation by MB-435 cells in a 3D model [63]. Using our system, we found that AIIB2 did not block cell multiplication of a tumorigenic cell line, but it did block coalescence and the formation of the three specialized cell types (probes, facilitators and dervishes). As shown by Weaver et al [13], it also inhibited subsequent aggregate development. The mAb, P1B5 against the subunit α-3 integrin, which can form a heterodimer with α-integrin and either ß-1 [58], did not block cell multiplication or coalescence. In fact, it enhanced the rate of coalescence. But most intriguingly, it caused a massive exodus of cells from the coalesced aggregates. Interestingly, Varzavand et al. [42] showed that down-regulating α-3 integrin using a RNAi-mediated silencing strategy in a prostate carcinoma cell line resulted in increased tumorigenesis, which is consistent with the exodus of cells in PIB5-treated aggregates. The limited results we present here for mAb-treated preparations demonstrate the specificity of the effects that can be attained for different agents using the 4D system described here. Discussing the role and interaction of the ß-1 and α-3 integrin subunits with the matrix and cellular markers, or the role of these integrin subunits in the regulation of the actin cytoskeleton and hence, the variety of cellular and multicellular processes integrins regulate, was not the intent of experiments presented here. Rather, the intent was to demonstrate the utility of the 4D system in analyzing the effects of drugs and mAbs on the formation of aggregation and coalescence.

### Further use of the model

The complexity of the 4D system described here may preclude it as a tool for large screen studies of potential anti-tumor drugs or mAbs. It does, however, provide a high resolution method for analyzing the cellular processes involved in coalescence and the effects of individual agents on these processes. It also will provide a framework for developing a more elaborate multi-cell type system for assessing the effects of the extracellular matrix and cells of the tumor microenvironment [64] such as immune cells [65,66] and fibroblasts [6], on the different aspects of aggregation and aggregate coalescence *in vitro*. In addition, the effects of targeted deletions of cancer relevant genes such as tumor suppressors [67] on these processes can also be studied. This will be especially valuable if, in fact, the processes we have identified *in vitro*, most notably the formation of putative specialized cell types, has correlates *in vivo*.

## Supporting Information

S1 Table Coalescence and the formation of the specialized cell types facilitator, probe and dervish, among cell lines and fresh cancer preparations. (PDF)

S1 Methods Automatic and manual 3D reconstruction. (PDF)

S2 Methods Computation of 3D parameters. (PDF)

S1 Fig Examples of automatic object detection by complexity-based bitmap object detection (C-BBOD), manual edge detection, and 3D reconstruction. A. DIC optical sections of aggregates. B. Autotraced optical sections of the in-focus portions of aggregates (purple areas) using C-BBOD. C. Adaptive skeleton climbing reconstructions of aggregates without smoothing. D. Vertex-smoothed reconstructions with DIC texture overlays. E. DIC optical sections of a single cell. F. Manual tracing of cell body (orange) and pseudopods (green). G. Adaptive skeleton climbing reconstruction of single cell without smoothing. H. Vertex-smoothed reconstruction with DIC texture overlay. See S1 Methods for details of methods.
(TIF)

S2 Fig Rotations of aggregate reconstructions presented originally in Fig. 2 and Fig. 3. A. Rotations at four select time points reinforce the conclusion from Fig. 2 that aggregates of the non-tumorigenic cell line MCF-10A do not coalesce. B. Rotations of MB-435-Br1 aggregates (from Fig. 3) at four select time points reinforces the conclusion from Fig. 3 that aggregates of the tumorigenic cell line MB-435-Br1 coalesce.
(TIF)

S3 Fig Inter-aggregate spaces in MCF-10A and MB-435-Br1 preparations reveal differences between the two. A. DIC images of optical sections of MCF-10A preparations revealed no cells in the inter-aggregative spaces. B. DIC images of optical sections of MB-435-Br1 preparations revealed cells in inter-aggregate spaces. Inter-aggregate spaces are encapsulated in a red line and cells are noted by red stars.
(TIF)

S4 Fig Quantitating number, motility and contour changes of aggregates as a function of time during the development in 3D reveals fundamental differences between the non-tumorigenic cell line (MCF-10A) and the tumorigenic cell line (MB-435-Br1) cell lines. A. Aggregation number in the area of analysis. B. Mean volume of aggregates in an area of analysis. C. Mean surface complexity of aggregates in an area of analysis. D. Mean speed of aggregate translocation. See S2 Methods for derivations of parameters.
(TIF)

S5 Fig The 3D path and 3D velocity of a reconstructed dervish cell in a MB-435-Br1 preparation. A. The 3D path plotted in an X, Y, and Z grid. B. The velocity of a dervish cell over an 18 hour period. Velocity was measured as described in S2 Methods.
(TIF)

S1 Movie J3D-DIAS 4.1 reconstructions of cells of the non-tumorigenic cell line MCF-10A cultured in 3D Matrigel reveal that aggregates increase in size due to cell division but do not coalesce and remain positionally fixed throughout the period of analysis. The movie covers 72 hours through 168 hours of culture.
(MOV)

S2 Movie In contrast to non-tumorigenic cells, aggregates of the tumorigenic cell line MoVi10 grow and then after 100 hours, move through the 3D Matrigel and rapidly coalesce within 3 days. MoVi10 is a tumorigenic cell line derived from a breast tumor (see S1 Table).
(MOV)

S3 Movie J3D-DIAS 4.1 reconstructions reveal that coalescence of aggregates in the tumorigenic cell line MB-435-Br1 is mediated by contact between a facilitator cell (green) with a filopod (yellow) and a probe cell (red). The probe exits the aggregate on the left, contacts the facilitator and the adhered cells begin to pull the two aggregates closer together in the process of coalescence.
(MOV)

S4 Movie J3D-DIAS 4.1 reconstructions of a dervish cell moving rapidly through the Matrigel in a swirling fashion. (MOV)
