# Supplementary material for: A Computer-Assisted 3D Model for Analyzing the Aggregation of Tumorigenic Cells Reveals Specialized Behaviors and Unique Cell Types that Facilitate Aggregate Coalescence
Source: PLoS One. 2015 Mar 19;10(3):e0118628. doi: 10.1371/journal.pone.0118628 (PMC4366230; doi:10.1371/journal.pone.0118628)
Supplement: S1 Table — (PDF) [file pone.0118628.s001.pdf]

S1 Table. Coalescence and the formation of the specialized cell types facilitator, probe and dervish, among cell lines and fresh cancer preparations.

Name	Type	Tissue	Coalescence	Cell types observed			Origin
				Facilitators	Probe	Dervish	
MCF-10A	cell line	human mammary epithelium	–	–	–	–	[1]
MARI 027	normal tissue	normal tissue lung	–	–	–	–	This study
MCF-7	cell line	breast cancer	-/+	–	+	–	[2]
MDA-MB-435-Br1	cell line	breast cancer derived	+++	+	+	+	[3, 4]
MDA-MB-435-α6HG6	cell line	breast cancer derived	+++	+	+	+	[3, 4]
MoVi10′	cell line	breast cancer	++++	–	+	–	[5]
LN18	cell line	glioblastoma	++	–	+	+	[6]
U87	cell line	glioblastoma	+++	+	+	+	[7, 8]
MARI 011	tumor tissue	omentum (breast cancer metastasis)	+++	–	+	–	This study
MARI 023	tumor tissue	kidney (paraanglioma)	+	+	+	–	This study
MARI 028	tumor tissue	lung tumor	++	–	+	+	This study
MARI M2	tumor tissue	melanoma	+++	+	+	+	This study

1. Soule, H.D., et al., *Isolation and characterization of a spontaneously immortalized human breast epithelial cell line, MCF-10.* Cancer research, 1990. **50**(18): p. 6075-6086.
2. Soule, H.D., et al., *A human cell line from a pleural effusion derived from a breast carcinoma.* J Natl Cancer Inst, 1973. **51**(5): p. 1409-16.
3. Baugher, P.J., et al., *Rac1 and Rac3 isoform activation is involved in the invasive and metastatic phenotype of human breast cancer cells.* Breast Cancer Res, 2005. **7**(6): p. R965-74.
4. Price, J.E., et al., *Tumorigenicity and metastasis of human breast carcinoma cell lines in nude mice.* Cancer research, 1990. **50**(3): p. 717-721.
5. Hendrix, M.J., et al., *Experimental co-expression of vimentin and keratin intermediate filaments in human breast cancer cells results in phenotypic interconversion and increased invasive behavior.* Am J Pathol, 1997. **150**(2): p. 483-95.

6. Diserens, A.C., et al., *Characterization of an established human malignant glioma cell line: LN-18.* Acta Neuropathol, 1981. **53**(1): p. 21-8.
7. Clark, M.J., et al., *U87MG decoded: the genomic sequence of a cytogenetically aberrant human cancer cell line.* PLoS Genet, 2010. **6**(1): p. e1000832.
8. Ponten, J. and E.H. Macintyre, *Long term culture of normal and neoplastic human glia.* Acta Pathol Microbiol Scand, 1968. **74**(4): p. 465-86.
