# Supplementary material for: A Computer-Assisted 3D Model for Analyzing the Aggregation of Tumorigenic Cells Reveals Specialized Behaviors and Unique Cell Types that Facilitate Aggregate Coalescence
Source: PLoS One. 2015 Mar 19;10(3):e0118628. doi: 10.1371/journal.pone.0118628 (PMC4366230; doi:10.1371/journal.pone.0118628)
Supplement: S1 Methods — (PDF) [file pone.0118628.s002.pdf]

S1 Methods: Automatic and manual 3D reconstruction.

Creating trace slots for each 3D object. A grayscale JPEG stack of 150 optical sections through the Z-axis was acquired over 2.0 minutes through an area of analysis using DIC microscopy and this process was repeated every 30 minutes for the present study. JPEG image compression is then applied. The software program J3D-DIAS 4.1 opens these large image stacks, bundles and indexes them to create a new internal file format native to this software program. The user enters two scale factors. The x, y scale factor (μm/pixel), measured from a stage micrometer, is a function of the total magnification. The z scale factor is the μm/level that the user determines before starting the acquisition of optical sections. The user then creates trace slots for individual cells, individual aggregates, a field of objects and filopodia. Two methods are then available for tracing these objects, automatic outlining and manual outlining. J3D-DIAS 4.1 was developed entirely in the W.M. Keck Dynamic Image Analysis Facility at the University of Iowa. It includes over 3 million lines of C and can be assessed by collaborative visits to the Facility.

Automatic outlining. Automatic identification of objects, which is based on the complexity of the image, was the method of choice for reconstructing aggregates. Complexity-based bitmap objection detection (C-BBOD) retains the interior of an object and is therefore useful for complex objects that may be hollow or contain gaps. It uses a complexity-based threshold algorithm. The complexity algorithm assigns a 0-256 gray scale value to individual pixels within a kernel. A kernel is a pixel matrix with a user-determined size (for example, 3x3, 5x5 or 7x7 pixels). The grayscale values of the pixels within the kernel are averaged, and the standard deviation (sd) of that average is

calculated and assigned to the reference pixel within the kernel. If the sd is greater than or equal to the user-determined threshold, the reference pixel is considered part of the object of interest and retained. The kernel is then moved by one pixel, calculations performed on a new reference pixel and the process repeated until every pixel within the entire frame has been analyzed. Complex objects such as cells or aggregates will exhibit a high degree of gray scale variation. Therefore, the pixels that comprise them will be retained. Background or images out of focus, on the other hand, will have near uniform grayscale values and will thus be discarded (i.e., not considered part of the object).

Manual edge detection. Manual outlining was used for single cells and fine structures such as filopodia. In this case, the gray scale threshold between an object and its background (i.e., the edge of the object) is determined by the user, who employs the computer mouse to trace and enter the outline of the object, as previously described in detail for 3D-DIAS in previous reports [1-7].

3D reconstruction. Upon completion of outlining, the tracings that overlap in the z-axis at each time point are grouped into a 3D z-series, either as a complexity stack (bitmap tracings) for autotraced objects or a series of outlines (beta-spline representations) for manually outlined objects. In the former case, the pixels are expanded into voxels (3D pixels) while in the latter, the outline is filled with voxels. In both cases, the voxel dimensions are determined by the x, y and z scales and the output is a 3D pixel map (raw voxel block). Next, the raw voxel blocks are wrapped to generate a continuous surface. Originally, 3D-DIAS employed a "tablecloth" method detailed elsewhere [8]. Briefly, in this method, a "tablecloth" is draped over the raw voxel blocks from the top down and from the bottom up and converted into a faceted surface. However, this method can

produce artifacts (vertical columns) in cases where the object extends over the surface but is not attached to the surface. Therefore, a variation of the "marching cubes" algorithm [9], known as the "adaptive skeleton climbing isoform extraction" algorithm [10], was introduced into J3D-DIAS4.1 and implemented using Java OpenGL (JOGL). Conceptually, adaptive skeleton climbing attaches a triangular facet of a user-determined size to the raw voxel block in the most stable possible configuration. The faceted surface of the entire object is then sequentially built upon this initial facet. Each added facet is placed in the most stable possible configuration. Cracks between facets are filled with irregularly shaped facets. The surface is then smoothed using a vertex smoothing algorithm [11]. In vertex smoothing, the x, y and z coordinates of a given vertex are averaged with neighboring x, y and z coordinates, respectively, to generate a new vertex that is usually slightly shifted in position, relative to the original one. Three rounds of vertex smoothing are performed. In this manner, irregularities or sharp edges in the surface of the object are attenuated.

One artifact inherent in vertex smoothing is that the object itself shrinks because the vertices tend to move towards each other. The artifact is negligible (~1%) in objects that are greater than 3,000 facets. Typical objects are 200,000 to 300,000 facets. In small objects less than 3,000 facets, the number of facets reaches 3,000. Optionally, the grays (i.e., textures) from the original DIC images in the raw voxel block can be mapped onto the facets to recreate a more life-like image. At this point, the image can be viewed from any angle in the Open GL module and calculations of contour and motility performed.

References

[1] Soll DR, Voss E, Johnson O, Wessels D (2000). Three-dimensional reconstruction and motion analysis of living, crawling cells *Scanning* **22**, 249-257.

[2] Soll DR, Wessels D, Heid PJ, Voss E (2003). Computer-assisted reconstruction and motion analysis of the three-dimensional cell *ScientificWorldJournal* **3**, 827-841.

[3] Soll DR, Wessels D, Voss E, Johnson O (2001). Computer-assisted systems for the analysis of amoeboid cell motility *Methods in molecular biology* **161**, 45-58.

[4] Wessels D, Kuhl S, Soll DR (2009). 2D and 3D quantitative analysis of cell motility and cytoskeletal dynamics *Methods in molecular biology* **586**, 315-335.

[5] Wessels D, Soll DR (1998). *Computer-assisted characterization of the behavioral defects of cytoskeletal mutants of Dictyostelium discoideum*. Soll D and Wessels D (eds). John Wiley, Inc. , pp. 101-140.

[6] Wessels D, Voss E, Von Bergen N, Burns R, Stites J, Soll DR (1998). A computer-assisted system for reconstructing and interpreting the dynamic three-dimensional relationships of the outer surface, nucleus and pseudopods of crawling cells *Cell motility and the cytoskeleton* **41**, 225-246.

[7] Wessels DJ, Kuhl S, Soll DR (2009). Light microscopy to image and quantify cell movement *Methods in molecular biology* **571**, 455-471.

[8] Soll D, Voss E (1998). *Two and three dimensional computer systems for analyzing how cells crawl.* . Soll D and Wessels D (eds). John Wiley, Inc. , pp. pp.25-52.

[9] Lorensen WE, Cline HE (1987). Marching cubes: A high resolution 3D surface construction algorithm *ACM SIGGRAPH Computer Graphics* **21**, 163-169.

[10] Poston T, Wong T-T, Heng P-A (1998). Multiresolution Isosurface Extraction with Adaptive Skeleton Climbing *Computer Graphics Forum* **17**, 137-147.

[11] Hermann L (1976). Laplacian-isoparametric grid generation scheme. *J Engin Mech Div* **102**, 749-756.
