# Supplementary material for: A Computer-Assisted 3D Model for Analyzing the Aggregation of Tumorigenic Cells Reveals Specialized Behaviors and Unique Cell Types that Facilitate Aggregate Coalescence
Source: PLoS One. 2015 Mar 19;10(3):e0118628. doi: 10.1371/journal.pone.0118628 (PMC4366230; doi:10.1371/journal.pone.0118628)
Supplement: S2 Methods — (PDF) [file pone.0118628.s003.pdf]

S2 Methods: Computation of 3D parameters.

A. Percent original aggregate number. To generate the plot of aggregate number over time, the number of aggregates was first counted in each 3D frame. To correct for the fact that aggregates can enter and exit the field of view at its edges due to shrinkage and distortion of the Matrigel, the number of aggregates in a field at the time of a merge (x) was divided by the total number of aggregates in the field just prior to the merge (y) and multiplied by 100, to get the "percentage original aggregate number".

B. Average volume per aggregate. The "average volume per aggregate" was calculated by J3D-DIAS 4.1 from faceted 3D reconstructions, which were mathematical models of the object [1]. The 3D position of the centroid (center) at each time point was computed by averaging the x-, y-, and z-coordinates of all interior points of the faceted object. The triangularized surface of the faceted object is a polyhedron. The calculus used to determine the volume of a polyhedron can be found at http://wwwf.imperial.ac.uk/~rn/centroid.pdf. The volumes of all objects in a field of view were then averaged for each time point and the averages plotted.

C. Surface complexity. Surface complexity was calculated by J3D-DIAS 4.1 from volume (described above) and surface area. The latter is the sum of the areas of all facets. The surface complexity parameter is a derivation of 3D roundness. The formula for the latter is

$$100\ \boldsymbol{x}\ 6\ \boldsymbol{x}\ \mathbf{sqrt}p\ \boldsymbol{x}\ \text{Vol}/(\text{surface area}^{3/2})$$

Where *sqrt* is the square root.

The result is a percentage relative to a perfect sphere, which gives a 3D roundness of 100%. Thus, the more complex the surface contour of an object is, the lower the 3D roundness parameter will be. However, to make the surface complexity measurement more intuitive, 3D roundness was expressed as the inverse rather than as a percentage of a perfect sphere. That is, surface complexity was computed as

$$1/(6\ \boldsymbol{x}\ \mathbf{sqrt}p\ \boldsymbol{x}\ \text{Vol}/(\text{surface area}^{3/2}))$$, where Vol is volume.

This yielded a value for surface complexity that increased, rather than decreased, with increasing complexity. To obtain the surface complexity graph, the surface complexity of every object in an area of analysis was averaged for each time point and plotted as a function of time.

D. <u>Speed of aggregate translocation.</u> The speed of aggregate translocation (instantaneous velocity) was calculated by the central difference method [2] using the same formulae applied to 2D objects [3] as follows:

$$\mathbf{Speed}[f] = (\mathbf{scale}\boldsymbol{x}\mathbf{frate})\ \boldsymbol{x}\ \mathbf{sqrt}\ (((\mathbf{x}[f+\mathbf{I}]-\mathbf{x}[f-\mathbf{I}])/2\mathbf{I}\ )^2 + ((\mathbf{y}[f+\mathbf{I}]-\mathbf{y}[f-\mathbf{I}])/2\mathbf{I}\ )^2 + ((\mathbf{z}f+\mathbf{I}]-\mathbf{z}[f-\mathbf{I}])/2\mathbf{I})^2\ )))$$
when $1<=f-\mathbf{I}$ and $f+\mathbf{I}<=\mathbf{F}$

$$\mathbf{Speed}[f] = (\mathbf{scale}\boldsymbol{x}\mathbf{frate})\ \boldsymbol{x}\ \mathbf{sqrt}\ (((\mathbf{x}[f+\mathbf{I}]-\mathbf{x}[f])/\mathbf{I})^2 + ((\mathbf{y}[f+\mathbf{I}]-\mathbf{y}[f])/\mathbf{I})^2 + ((\mathbf{z}f+\mathbf{I}]-\mathbf{z}[f-\mathbf{I}])/2\mathbf{I})^2\ )))$$
when $f-\mathbf{I}< 1$ and $f+\mathbf{I}<=\mathbf{F}$ (first frame)

$$\mathbf{Speed}\ [f] = (\mathbf{scale}\boldsymbol{x}\mathbf{frate})\ \boldsymbol{x}\ \mathbf{sqrt}\ (((\mathbf{x}[f]-\mathbf{x}[f-\mathbf{I}])/\mathbf{I})^2 + ((\mathbf{y}[f]-\mathbf{y}[f-\mathbf{I}])/\mathbf{I})^2 + (\ (\mathbf{z}f+\mathbf{I}]-\mathbf{z}[f-\mathbf{I}])/2\mathbf{I})^2\ )))$$
when $1<=f-\mathbf{I}$ and $f+\mathbf{I}>\mathbf{F}$ (last frame),

where **F** is the total number of frames,

f is the ‘current’ frame,

(**x**[f],**y**[f]) are the coordinates of the centroid of an object in frame f, 1<=f<=**F**,

I is the centroid increment,

frate is the frame rate in # of frames per unit time,

scale is the scale factor in distance units per pixel,

sqrt is the square root function, and

x denotes multiplication.

Speed [f] = 0

Using this method, the instantaneous velocity (speed of aggregate translocation) was calculated by J3D-DIAS 4.1 for each 3D aggregate. The instantaneous velocity of every object in each area of view was averaged for each time point and plotted.

References

[1] Soll D, Voss E (1998). *Two and three dimensional computer systems for analyzing how cells crawl.* . Soll D and Wessels D (eds). John Wiley, Inc. , pp. pp.25-52.

[2] Maron MJ (1982). *Numerical Analysis*, Macmillan: New York.
[3] Soll DR (1995). The use of computers in understanding how animal cells crawl *Int Rev Cytol* **163**, 43-104.
