# Supplementary figures and images for: A Computer-Assisted 3D Model for Analyzing the Aggregation of Tumorigenic Cells Reveals Specialized Behaviors and Unique Cell Types that Facilitate Aggregate Coalescence
Source: PLoS One. 2015 Mar 19;10(3):e0118628. doi: 10.1371/journal.pone.0118628 (PMC4366230; doi:10.1371/journal.pone.0118628)

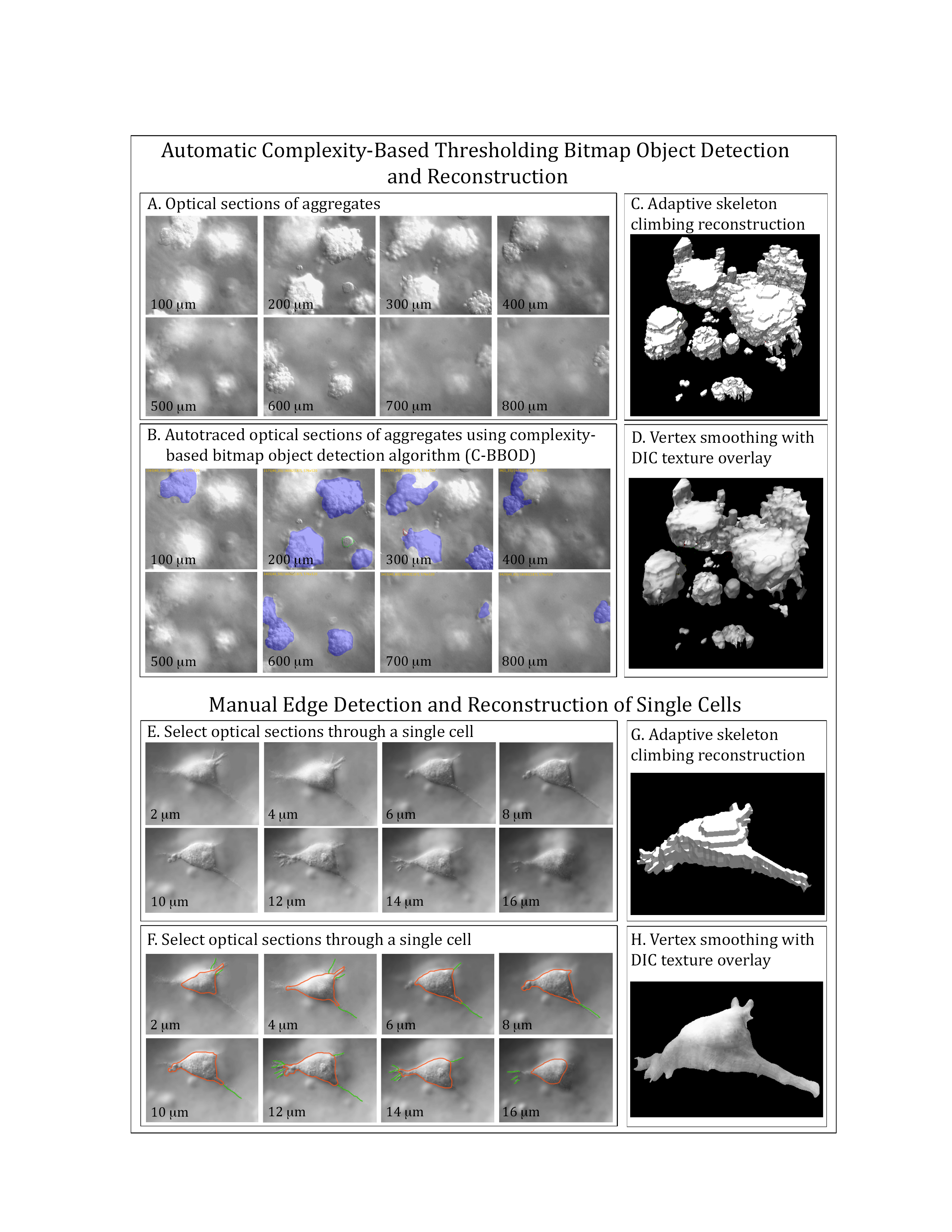

Supplement: S1 Fig — A. DIC optical sections of aggregates. B. Autotraced optical sections of the in-focus portions of aggregates (purple areas) using C-BBOD. C. Adaptive skeleton climbing reconstructions of aggregates without smoothing. D. Vertex-smoothed reconstructions with DIC texture overlays. E. DIC optical sections of a single cell. F. Manual tracing of cell body (orange) and pseudopods (green). G. Adaptive skeleton climbing reconstruction of single cell without smoothing. H. Vertex-smoothed reconstruction with DIC texture overlay. See S1 Methods for details of methods. (TIF) [file pone.0118628.s004.tif]

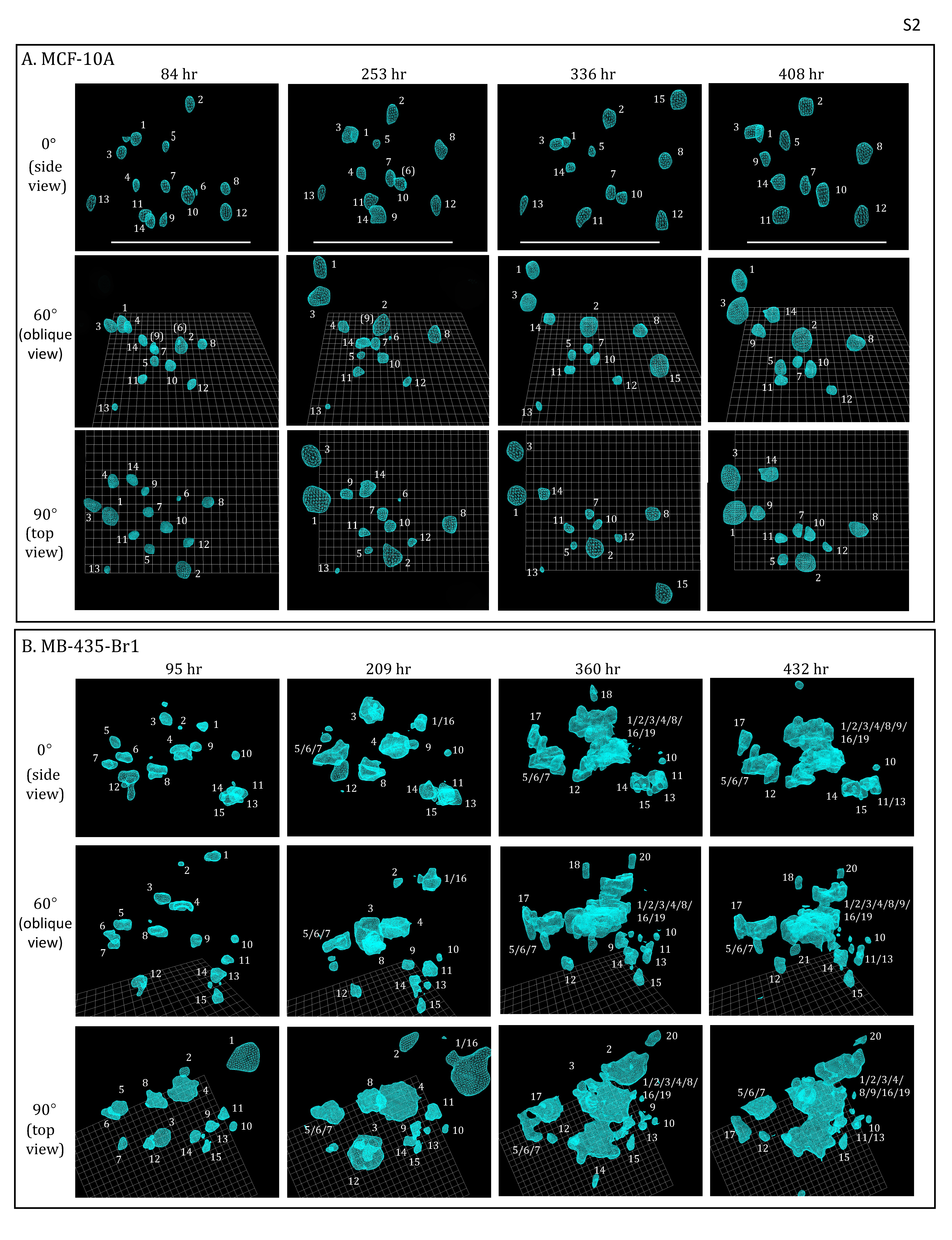

Supplement: S2 Fig — A. Rotations at four select time points reinforce the conclusion from Fig. 2 that aggregates of the non-tumorigenic cell line MCF-10A do not coalesce. B. Rotations of MB-435-Br1 aggregates (from Fig. 3) at four select time points reinforces the conclusion from Fig. 3 that aggregates of the tumorigenic cell line MB-435-Br1 coalesce. (TIF) [file pone.0118628.s005.tif]

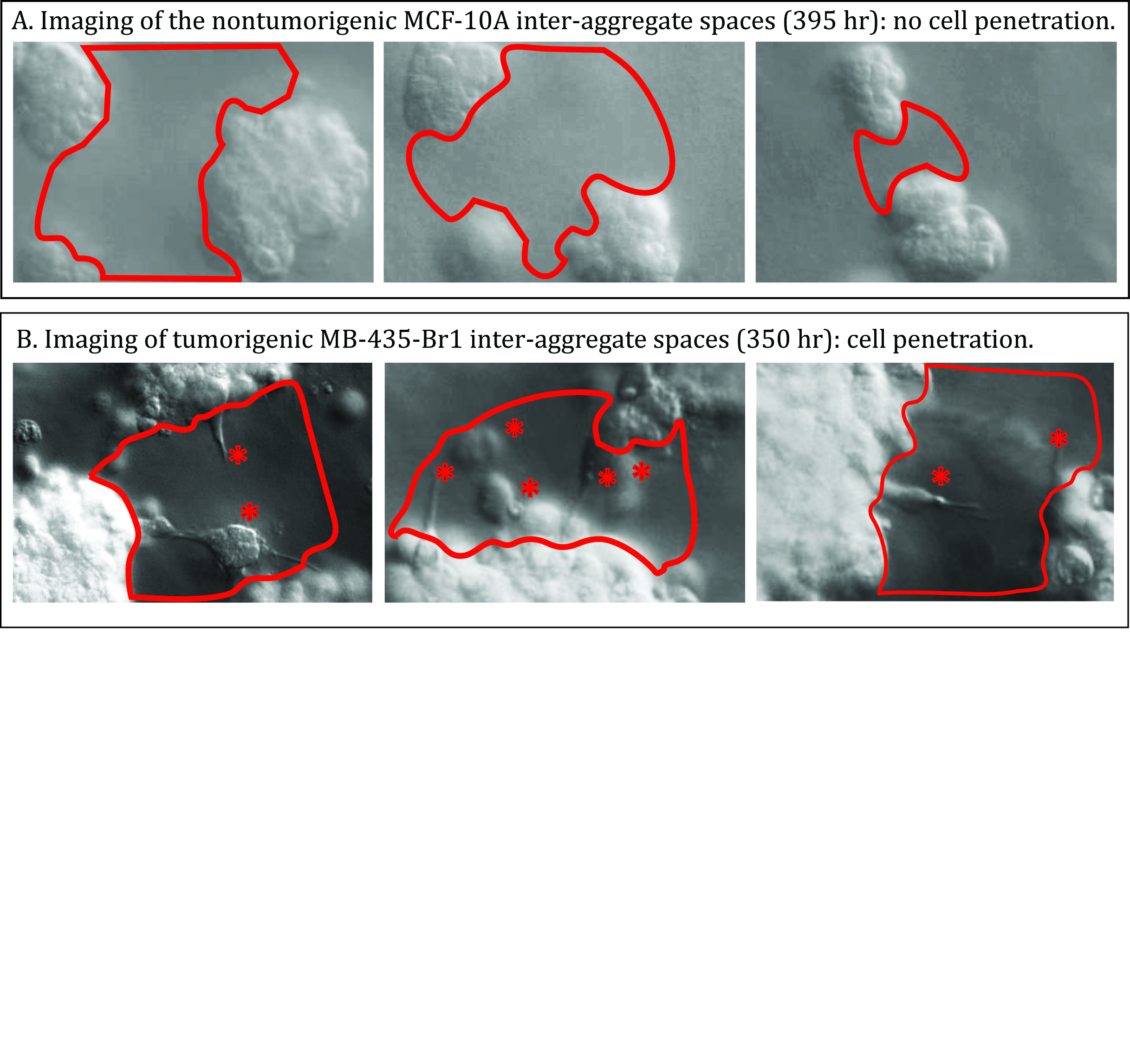

Supplement: S3 Fig — A. DIC images of optical sections of MCF-10A preparations revealed no cells in the inter-aggregative spaces. B. DIC images of optical sections of MB-435-Br1 preparations revealed cells in inter-aggregate spaces. Inter-aggregate spaces are encapsulated in a red line and cells are noted by red stars. (TIF) [file pone.0118628.s006.tif]

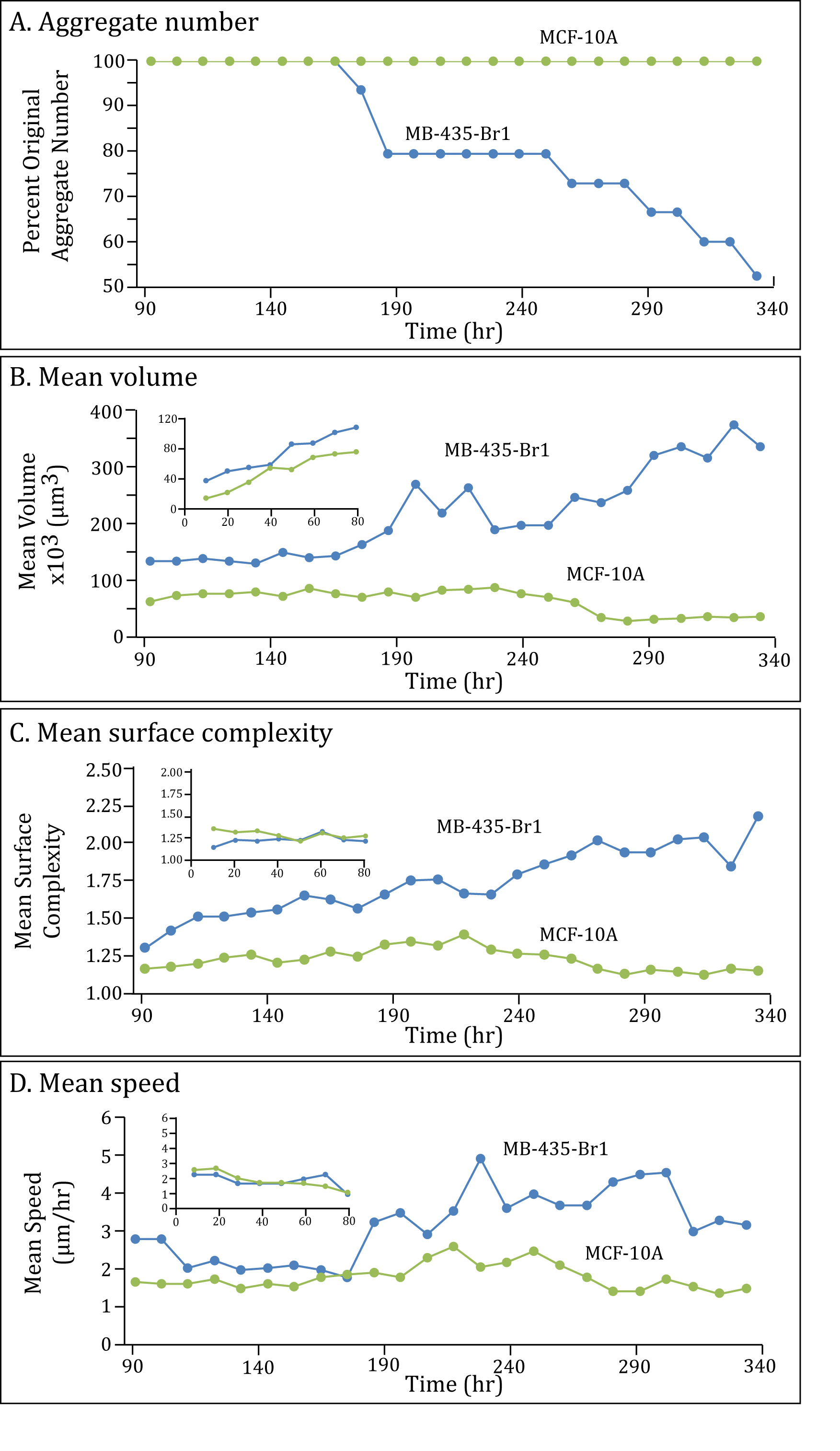

Supplement: S4 Fig — A. Aggregation number in the area of analysis. B. Mean volume of aggregates in an area of analysis. C. Mean surface complexity of aggregates in an area of analysis. D. Mean speed of aggregate translocation. See S2 Methods for derivations of parameters. (TIF) [file pone.0118628.s007.tif]

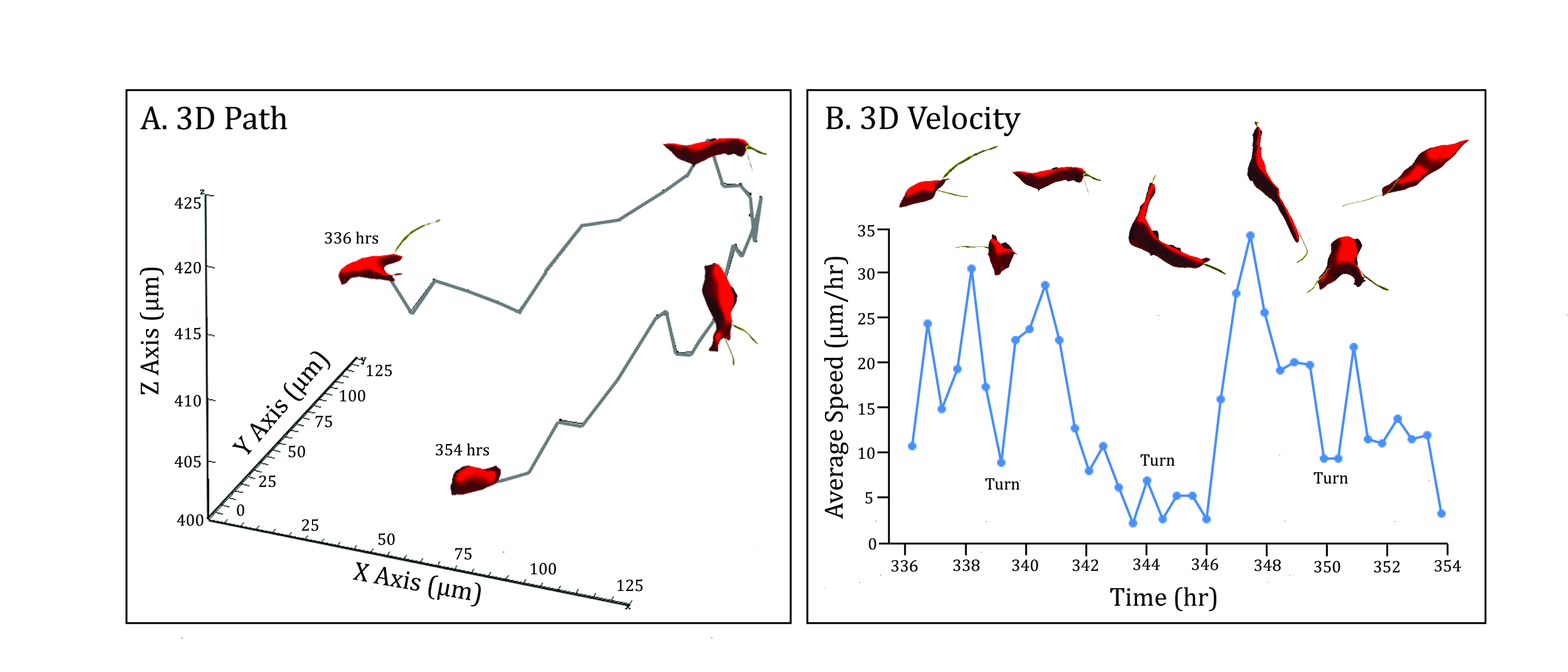

Supplement: S5 Fig — A. The 3D path plotted in an X, Y, and Z grid. B. The velocity of a dervish cell over an 18 hour period. Velocity was measured as described in S2 Methods. (TIF) [file pone.0118628.s008.tif]
